# Colloidal Quasi‐2D Cs_2_AgBiBr_6_ Double Perovskite Nanosheets: Synthesis and Application as High‐Performance Photodetectors

**DOI:** 10.1002/smll.202513500

**Published:** 2026-02-09

**Authors:** Pannan I. Kyesmen, Eugen Klein, Brindhu Malani S, Rostyslav Lesyuk, Christian Klinke

**Affiliations:** ^1^ Institute of Physics University of Rostock Rostock Germany; ^2^ Pidstryhach Institute for Applied Problems of Mechanics and Mathematics of NAS of Ukraine Lviv Ukraine; ^3^ Department Life Light & Matter University of Rostock Rostock Germany

**Keywords:** colloidal synthesis, detectivity, double perovskites, photodetection, quasi‐2D nanosheets, response time

## Abstract

The search for non‐toxic lead‐free halide perovskites that can compete with the lead‐based counterparts has led to the emergence of double perovskites as potential candidates. Among many options, Cs_2_AgBiBr_6_ stands out as one of the most suitable eco‐friendly materials for numerous optoelectronic applications. In this study, quasi‐2D Cs_2_AgBiBr_6_ nanosheets (NSs) were prepared via the low‐temperature injection colloidal synthesis and used to fabricate high‐performance photodetectors in a transport‐layer‐free architecture. The reaction temperature and ligands played vital roles in the structural purity, shape, and size of the synthesized Cs_2_AgBiBr_6_ NSs. The fabricated NSs disclosed lateral sizes of up to 1.4 µm and are only a few nanometers thick. The high‐performance photodetectors fabricated using the Cs_2_AgBiBr_6_ NSs yielded a high detectivity (*D*) of 1.15 × 10^12^ Jones, responsivity (*R*) of 121 mA/W, a notable on‐off ratio of 2.39 × 10^4^, and a fast rise and decay time of 857 and 829 µs, respectively. The device demonstrates remarkable stability. Basically, it sustains its entire photocurrent after storage in ambient conditions for 80 days. This work showcases a pathway for the colloidal synthesis of quasi‐2D Cs_2_AgBiBr_6_ lead‐free double perovskite NSs with suitable properties for high‐performance photodetection and other optoelectronic applications.

## Graphical abstract

Layered quasi‐2D Cs_2_AgBiBr_6_ nanosheets, with large lateral dimensions of up to 1.4 µm, which are only a few nanometers thick, were synthesized as a result of systematic investigation of the roles of ligands and reaction temperature. The nanosheets were used to develop high‐performance photodetectors, yielding detectivity in the order of 10^12^ Jones with response times in the sub‐millisecond region.

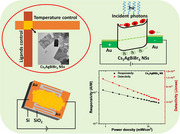

## 1 Introduction

The difficulty in managing lead (Pb) toxicity and stability issues in Pb halide perovskites has steered an intense search for stable and lead‐free perovskites that can yield comparable properties to their Pb‐based counterparts for many optoelectronic applications. The earlier solutions attempted to directly replace Pb in the APb^2+^X_3_ basic composition with a similar divalent cation such as Sn and Ge. For Sn, the huge number of defects and stability issues arising from the easy oxidation of Sn^2+^ to Sn^4+^ under ambient conditions poses a major challenge for their application in optoelectronic devices [1]. On the other hand, Ge is a rare metal, and its scarcity makes it very expensive and unattractive for practical applications in photovoltaic and optoelectronic devices [2]. Moreover, it also suffers from stability issues and can easily oxidize from Ge^2+^ to Ge^4+^ [3]. The emergence of double perovskites has opened promising options for developing low‐cost and environmentally friendly halide perovskites for photovoltaic and optoelectronic applications. The heterovalent replacement of two divalent Pb^2+^ ions in Pb‐based perovskites with one monovalent ion (Na^+^, Ag^+^, Cu^+^) and one trivalent ion (Bi^3+^, In^3+^, Sb^3+^) forms a diverse class of promising quaternary A_2_M^+^M^3+^X_6_ double perovskites. This configuration ensures charge neutrality similar to conventional lead‐based perovskites, structural diversity, and controllable size, shape, and electronic band properties for a more stable material [4].

Many Pb‐free double perovskites, including Cs_2_AgInCl_6_ [5] and Na_2_CuBiBr_6_ [6], have been developed after the first reports of Cs_2_AgBiBr_6_ bulk crystals in early 2016 [7, 8]. Despite these options, Cs_2_AgBiBr_6_ is still one of the most suitable eco‐friendly materials for numerous optoelectronic applications. While Pb‐based perovskites are still investigated for many applications, including photodetection [9, 10, 11], Cs_2_AgBiBr_6_ has shown great potential to serve as an environmentally‐friendly alternative for many applications. The material possesses desirable properties such as long carrier lifetimes, high chemical, thermal, and ambient stability, relatively lower effective mass of charge carriers, insignificant toxicity, and high light absorption coefficient [12, 13], beneficial for various optoelectronic applications. When prepared as confined 2D/quasi‐2D structures, they offer the unique advantage of quantum confinement effects and wide bandgap tunability, which can be relevant for effective engagement in applications such as photovoltaics, photoelectrochemistry, and photodetection.

Photodetectors based on 2D/quasi‐2D materials are among the most competitive candidates for efficient and cost‐effective device design. This is because 2D/quasi‐2D materials are typically synthesized in their single‐crystalline states and have fewer ionic defects and grain boundaries when compared to their corresponding 3D polycrystalline films [14]. Moreover, when in confinement, 2D/quasi‐2D materials exhibit intense absorbance at specific wavelengths that permit optical tunability and wavelength‐selective photodetection applications. 3D materials of Cs_2_AgBiBr_6_ double perovskites have exhibited promising photodetection properties [15, 16, 17]. However, the presence of unavoidable defects and a huge level of grain boundaries in 3D polycrystalline particles results in large photocurrent hysteresis, reduced photostability and moisture tolerance, and decreased performance reliability [14, 18]. Meanwhile, 2D Cs_2_AgBiBr_6_ double perovskites exhibit low intrinsic carrier density and high resistivity in the out‐of‐plane direction, which leads to low dark current and consequently high detectivity. This has been demonstrated in a previous study where 2D Cs_2_AgBiBr_6_ fabricated using the space‐confined technique yields superior photodetection properties over their 3D counterparts [13]. Typical 2D perovskites form bound excitons with a higher binding energy when excited, which limits conductivity (in the out‐of‐plane direction) and charge transport in photodetection applications [18]. However, quasi‐2D perovskites can harness the benefits of both 2D and 3D structures for better device design and performance, which has been demonstrated in many applications, including photovoltaic [19], supercapacitors [20], and photodetection [21]. The quantum confinement effects, high surface‐to‐volume ratio, high absorption coefficient, and out‐of‐plane high resistivity make 2D/quasi‐2D Cs_2_AgBiBr_6_ attractive for efficient photodetection design for practical applications.

The ball milling approach [22], space‐confined fabrication [13], and colloidal synthesis [23] are among the techniques that have been engaged for the preparation of confined 2D/quasi‐2D nanostructures of Cs_2_AgBiBr_6_. The colloidal synthesis offers a versatile, low‐cost approach for preparing high‐quality, quantum‐confined single crystals of Cs_2_AgBiBr_6_. In 2021, Liu et al. developed a low‐temperature injection colloidal technique for the first synthesis of quantum‐confined 2D Cs_2_AgBiBr_6_ double perovskite nanoplatelets [24]. Further work done in 2023 by Dor et al. provides additional insight into the growth mechanism of the nanoplatelets and proposed self‐assembly of small platelets into larger ones during the crystal formation, which results in some physical defects [25]. In both cases, the confined nanoplatelets were prepared at a reaction temperature of 230°C, and consisted of nanoplatelets with a lateral size of 330 ± 230 nm and a thickness of 3.6–6.0 nm. These studies did not provide a comprehensive report on the role of reaction temperature and ligands in the growth of colloidal Cs_2_AgBiBr_6_ nanocrystals. More so, the successful colloidal synthesis of Cs_2_AgBiBr_6_ nanoplatelets/nanosheets above 230°C has not been reported based on known literature. In colloidal synthesis, the reaction temperature and ligands can significantly influence the size, shape, structural defects, and crystallization of nanomaterials. In the colloidal synthesis of perovskite nanocrystals, an increase in the concentration of ligands can provide a unique acid‐base equilibrium that could allow their crystallization at a higher temperature, thereby influencing their size, shape, and structure [26].

In this project, colloidal quasi‐2D Cs_2_AgBiBr_6_ nanosheets (NSs) were fabricated using a low‐temperature injection and heat‐up process, and the role of reaction temperature and ligands in the synthesis was systematically investigated. The Cs_2_AgBiBr_6_ NSs were used to develop high‐performance photodetectors. Quasi‐2D Cs_2_AgBiBr_6_ NSs with a lateral size of up to 1.4 µm and only a few nm thick were fabricated at 250°C reaction temperature under favourable ligand conditions, without distorting the material's crystal structure. The reaction temperature and ligands served crucial roles in the structural integrity, shape, and size of the synthesized Cs_2_AgBiBr_6_.The high‐performance photodetectors recorded a high detectivity of 1.15 × 10^12^ Jones, responsivity of 121 mA/W, and fast rise and fall times of 857 and 829 µs, respectively. In addition, the device recorded a notably high on‐off ratio of 2.39 × 10^4^. It is worth noting that the Cs_2_AgBiBr_6_ photodetectors attained such high performance in a charge transport layer‐free architecture, which usually adds to the complexity of device design and cost effectiveness. This work paves a pathway for developing large quantum‐confined NSs of colloidal Pb‐free Cs_2_AgBiBr_6_ double perovskites with suitable properties for high‐performance photodetection and other optoelectronic applications.

## 2 Results and Discussion

### 2.1 Cs_2_AgBiBr_6_ Nanosheets

Nanosheets of Cs_2_AgBiBr_6_ double perovskites were synthesized using the low‐temperature crystallization approach first developed by Liu et al. in 2021, with some modifications to the final reaction temperature and amount of oleic acid (OA) ligand used in the synthesis [24]. An increase in the amount of OA (from 1.0 to 1.5 mL) allowed for the synthesis of Cs_2_AgBiBr_6_ NSs at a higher final reaction temperature of 250°C (instead of 230°C), allowing for the synthesis of laterally larger NSs without any structural distortion. In brief, silver nitrate (AgNO_3_), bismuth bromide (BiBr_3_), 1‐octadecene (ODE), OA, oleylamine (OLAm), and hydrobromic acid (HBr) were added into a three‐necked flask, degassed at 120°C, and heated to 200°C under continuous Ar flow to ensure that the mixture is fully dissolved. The solution was cooled to room temperature, followed by the injection of Cs‐oleate and stirring for 10 min. The mixture was then heated to 250°C for 10 min to obtain NSs of Cs_2_AgBiBr_6_. The growth mechanism of the nanostructures through the self‐assembly of smaller platelets into larger ones has been proposed and explained in a previous study [25]. Given appropriate reaction conditions and a higher reaction temperature, the small nanoplatelets will acquire more thermal and kinetic energy, causing them to migrate faster, increase their reactivity, and yield larger NSs. The roles that ligands and temperature play in preparing quantum‐confined NSs of Cs_2_AgBiBr_6_ double perovskites are investigated and will be discussed in more detail in Section 2.2.

**FIGURE 1:**
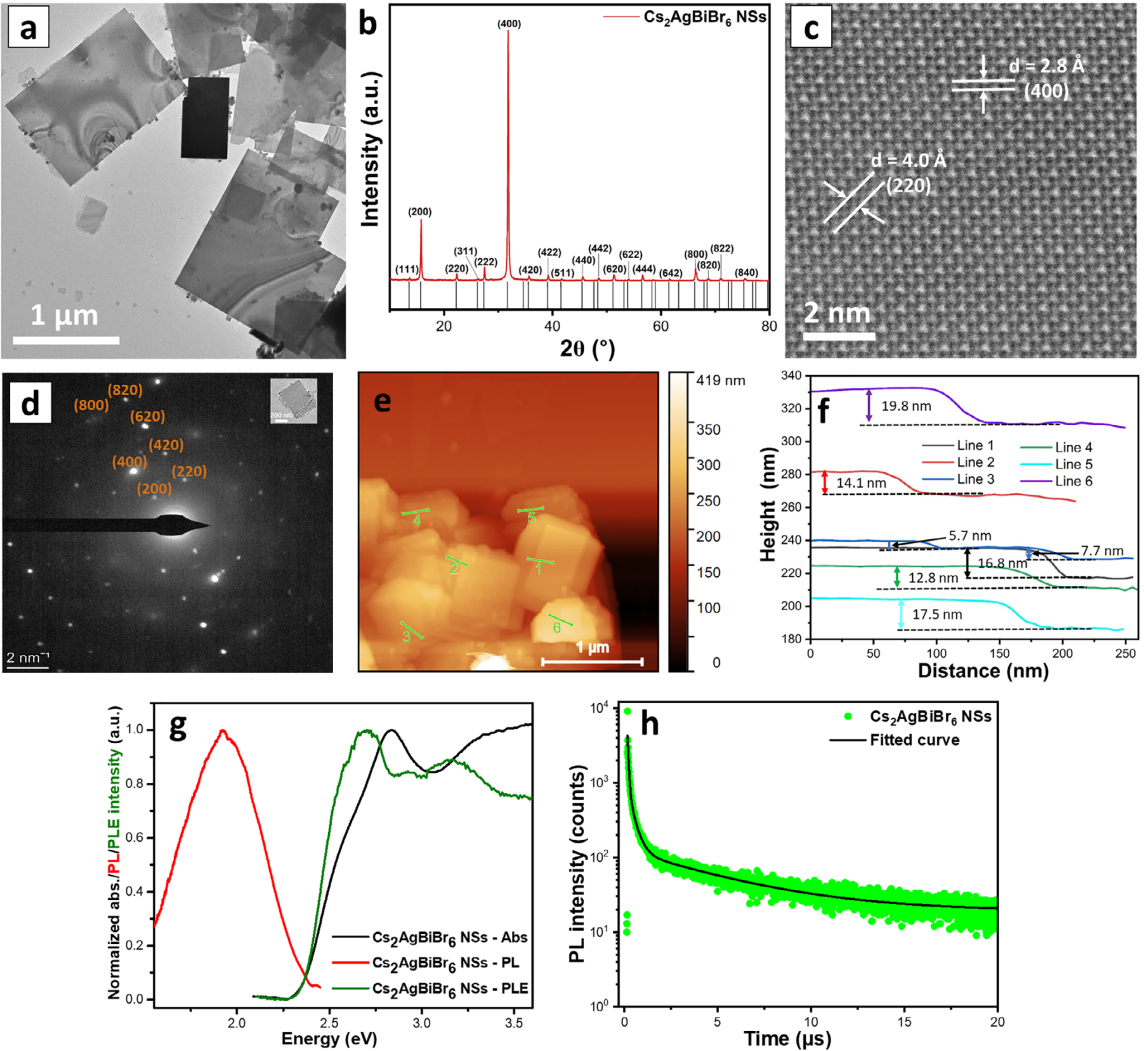
The synthesized Cs_2_AgBiBr_6_ NSs. (a) TEM micrograph, (b) XRD pattern (cubic phase of Cs_2_AgBiBr_6_ according to ICSD card number 01‐087‐9014), (c) HRTEM image, (d) SAED pattern, (e) AFM image showing NSs and (f) their height profiles, (g) UV–vis absorption, PL, and PLE spectra, (h) time‐resolved PL decay and its corresponding fitted curve.

Figure 1a shows the transmission electron microscopy (TEM) image of the synthesized Cs_2_AgBiBr_6_ NSs (Figure S1a shows a more expanded view at a lower magnification). The micrograph revealed NSs with rectangular shapes and a wide lateral size distribution of 0.3–1.4 µm. The maximum lateral size of 1.4 µm observed for the NSs is way more than double the highest lateral dimension previously obtained for the colloidal nanoplatelets of the material [24, 25]. For statistical clarity, the histogram for the lateral size distribution of the NSs yielded a mean size of 0.73 µm with a standard deviation (SD) of 0.23 (inset of Figure S1a). The lateral growth of the nanoplatelets into larger NSs was induced by an increase in the quantity of OA in the reaction solution, which provided a suitable acid‐base equilibrium for the ligands and enabled the appropriate shelling of the platelets that allowed for the reaction to be sustained at 250°C without distorting the structure and composition of the material. Higher reaction temperature increases the thermal energy in the system, which enhances the migration and growth of smaller platelets into NSs. For application of Cs_2_AgBiBr_6_ as photodetectors, NSs with higher lateral dimensions will effectively bridge the electrode spacing and reduce grain boundaries, which can serve as traps for charge carriers, thereby promoting charge transport and device performance [27, 28]. X‐ray diffraction (XRD) measurements conducted on the fabricated NSs showed patterns that emphatically aligned with the cubic phase of the crystal structure of Cs_2_AgBiBr_6_ according to ICSD card number 01‐087‐9014 and exhibited an orientation in the <001> direction when deposited on the substrates, as seen in the high intensity observed for (400) and (200) planes (Figure 1b). The low‐angle diffraction patterns revealed periodic peaks, which indicate that the NSs are stacked along the <001> direction (Figure S1b). The periodicities of 4.0 and 3.5 nm were deduced from equidistant reflections in the low‐angle XRD patterns, which reveal a stacking behavior of single ultrathin nanosheet layers to form the quasi‐2D nanostructures. The difference between these two values is approximately equal to a half unit cell of the Cs_2_AgBiBr_6_ (∼0.57 nm, called further as monolayer (ML)), meaning that the thickness of individual 2D layers increases by one ML of [Ag, Bi]Br octahedrons. The size of a unit cell was calculated from the XRD data using Bragg's law and the Miller indices (Table S1 and Section SI1). Considering the length of OLAm to be ∼1.7 nm [25], the thickness of the individual nanosheet layer is then estimated to be ∼1.8 nm in the first case and ∼2.3 nm in the second case. The high‐resolution TEM (HR‐TEM) performed on a single nanosheet of Cs_2_AgBiBr_6_ further affirms good crystallinity with clear lattice fringes over the whole sheet (Figure 1c). Lattice d‐spacings of 2.8 and 4.0 Å were calculated from the lattice fringes and assigned to the (400) and (220) of the cubic structure of bulk Cs_2_AgBiBr_6_. The fast Fourier transform (FFT) pattern derived from the HR‐TEM analysis further affirms the material's cubic structure (Figure S2). Selected area electron diffraction (SAED) analysis conducted on a single sheet also shows growth along the [00$\bar{1}$] zone axis (Figure 1d) for the cubic crystal of Cs_2_AgBiBr_6_. The lattice spacing calculated from the observed planes in the SAED image matches well with the values calculated from the XRD pattern of the Cs_2_AgBiBr_6_ NSs, as given in Table S1. TEM/energy dispersive X‐ray spectroscopy (TEM‐EDS) measurements detected signals for all the elements of the NSs with an atomic weight ratio of 1.9:1.0:1.1:6.8 for Cs:Ag:Bi:Br in line with the expected 2:1:1:6 stoichiometry of the material (Figure S3a). EDS elemental mapping done on Cs_2_AgBiBr_6_ NSs (HR‐TEM micrograph shown in Figure S3b) revealed a uniform distribution of the constituent elements across the material's surface (Figure S3c–f). Atomic force microscopy (AFM) analysis revealed stacked NSs that are 5.7–19.8 nm thick (Figure 1e,f). The thinnest structures of ∼5.7 nm align well with the consideration of two OLAm layers with an inorganic part of ca. 2 nm. The thickness of individual ultra‐thin layers would then correspond to about 1.5 unit cells or 3 [Ag, Bi]Br ML, and the thickness of 2.3 nm would correspond to 4 ML.

The absorption spectrum of the Cs_2_AgBiBr_6_ NSs is given in Figure 1g and revealed an excitonic absorption peak centered at 2.83 eV (437.8 nm), which is near the band edge, and attributed to the 6s–6p transition of Bi in the [BiBr_6_]^3–^ octahedra of the material's crystal structure [29]. The absorption at higher energies with a peak at 3.40 eV (365.1 nm) results from direct bandgap absorption, similar to previous reports [3, 16]. A very weak absorbance feature is observed at about 2.55 eV, which we attribute to excitations from the few occurrences of NSs with a high number of stacked layers (Figure 1a; Figure S1a) and bulk‐like nanostructure's properties. The NSs exhibited broad PL with an emission peak position at 1.92 eV (644.8 nm), a FWHM of 7.75 eV (159.9 nm), and a redshift of 6.0 eV (207.0 nm) with respect to the excitonic absorption peak. The broad emission spectrum and large Stokes shift are considered to be a result of strong electron–phonon coupling and emission from self‐trapped excitons (STEs), which have been widely discussed for different types of perovskites [30, 31]. PL excitation (PLE) measurements were conducted on the NSs to better understand the broad emission observed for the material (Figure 1g). The PLE shows two interesting features at ∼120 and 92 meV below and above the absorption resonance at 2.83 eV. Such energy splitting of ∼50–60 meV above/below the resonance absorption has been observed for Cs_2_AgBiBr_6_ thin films in a previous study and investigated using low‐temperature studies and several spectroscopic techniques. It is proposed that the transitions at higher/lower energies relative to the resonance absorption have lower oscillator strengths, making relaxation to the ground state more favourable, thereby forming the two distinct peaks observed in the PLE spectra. Meanwhile, the higher oscillation strength of the transition at 2.83 eV allowed it to become more dominant in the optical absorption spectrum [32]. The excited states' relaxation to the ground state involves a slow and radiative process that results in the broad and highly redshifted PL emission observed for the material, and the long decay component later seen in time‐resolved PL (TRPL). The PLE peak observed at 2.71 eV appears to be broadened, and this is associated with contributions related to the weak transition observed at about 2.55 eV in the absorption spectra due to excitations from limited occurrences of bulk‐like NSs in the material. The room temperature TRPL decay of the NSs was fitted to a tri‐exponential function, which produced a fast and intermediate component with lifetimes of 55.1 and 316.8 ns, respectively, and a slow decay of 4.8 µs (Figure 1h). The fitting details are given in Table S2. The relatively faster and intermediate decay processes are attributed to the recombination processes due to surface defects and trap states. The slow component is associated with radiative recombination processes linked to self‐trapped exciton states, which are assumed to be easily depopulated by structural defects [7, 31].

### 2.2 Role of Temperature and Ligands

**FIGURE 2:**
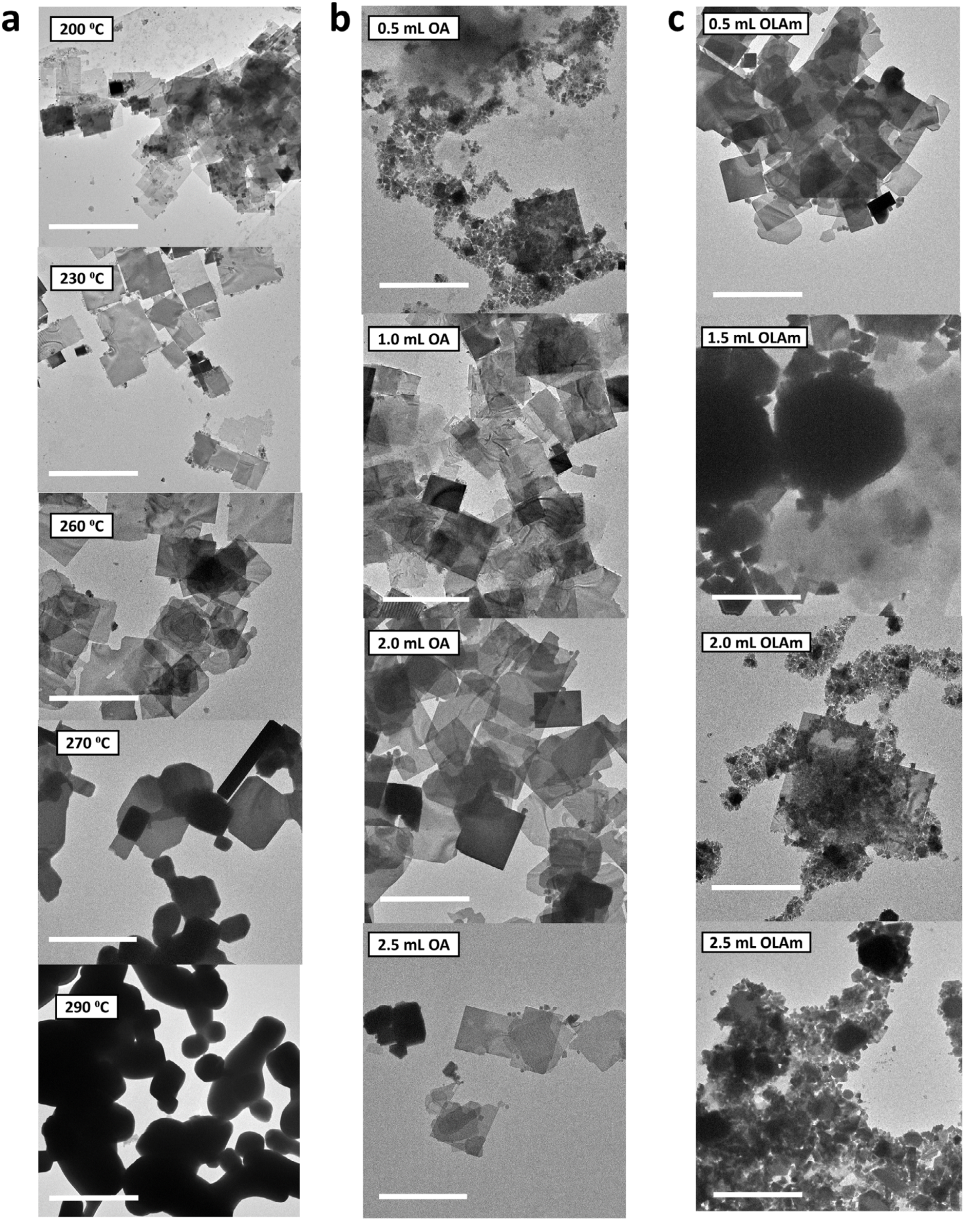
TEM images of colloidal Cs_2_AgBiBr_6_ samples prepared (a) at different final reaction temperatures, and using various concentrations of (b) OA and (c) OLAm, respectively. The scale bars shown in the micrographs represent 1 µm. For clarity, refer to Figure 1a for the TEM image of the sample prepared at the reaction temperature of 250°C, which contains 1.5 and 1.0 mL of OA and OLAm in the reaction mixture, respectively.

The role of the final reaction temperature in the colloidal synthesis of Cs_2_AgBiBr_6_ double perovskites on their structural and optical characteristics was investigated. Figure 2a shows the TEM image of the samples prepared at final reaction temperatures ranging from 200°C to 290°C. The samples prepared at 200°C disclosed small nanoplatelets and some large sheets with lateral dimensions ranging from ∼10–500 nm. The lateral size of the nanoplatelets increases with the reaction temperature up to 250°C, where nanosheets with a maximum lateral size of 1.4 µm were obtained. As mentioned before, this enhanced lateral growth is due to an increase in the thermal energy in the system at such a high‐temperature, which promotes the migration and growth of smaller nanoplatelets into larger sheets. At the final reaction temperature of 260°C, the nanosheets began to deform and eventually produced bulk‐like particles at 290°C. This deformation is due to high reactivity at elevated temperatures that causes instability in the shelling of the nanoplatlets by the ligands, leading to inhomogeneous crystal growth, formation of byproducts, and eventual growth into bulk‐like crystals. The XRD (Figure S4a) affirms the good quality of the crystals prepared at final reaction temperatures between 200°C –250°C, as all the peaks observed clearly matched the cubic phase of Cs_2_AgBiBr_6_ double perovskites. Extra XDR peaks were observed for samples prepared at 260°C and above, at 2θ values of 30.95°, 44.34°, and 50.08°, which are associated with the (200), (220), and (222) planes of the cubic crystal structure of AgBr, respectively (ICSD card number: 01‐071‐4692). Furthermore, the intensity of the XRD peaks along the z‐direction increases significantly, especially for samples prepared at 270–290°C, which affirms the growth of the NSs to bulk‐like particles. The UV–vis absorption spectra show confined excitonic excitation peaks around 2.83 eV (expected to be near the band edge) for samples prepared between 200°C –250°C. The exciton absorption peaks were broadened and redshifted by 0.06 and 0.11 eV for the samples prepared at 270 and 290°C, respectively, implying a change toward bulk‐like nanostructures of Cs_2_AgBiBr_6_. Further extending the final reaction time beyond the normal 10 min (up to 60 min) also resulted in the formation of byproducts and eventual growth toward bulk‐like particles after 60 min, which is also due to unstable ligand shells around the platelets and excessive reactivity in the system (Figure S5a,b). The excitonic absorption peaks became broadened and redshifted by 0.02 and 0.07eV when the final reaction time was increased to 20 and 60 min, respectively, which indicates a decrease in quantum confinement (Figure S5c). All the samples prepared under different reaction temperature and time conditions exhibited broad PL emission at similar wavelengths of 644.8 ± 0.04 nm (Figures S4c and S5d). For statistical purposes, the histograms for the lateral size distribution, including the statistical details of mean size and SD of the NSs prepared at reaction temperatures of 200°C, 230°C, and 260°C, are given in Figure S6a–c and discussed in Section SI2. The TEM images of the samples prepared at reaction temperatures of 270°C and 290°C (Figure 2a) and higher reaction times of 30 and 60 min (Figure S5a) were not suitable for size distribution analysis because of the deformation in the shape of the nanostructures that results from the high reactivity in the reaction system.

Another crucial factor in the colloidal synthesis of nanostructures is the role ligands play in controlling their shape, size, uniformity, and structural defects. The role of key ligands – OA and OLAm, used in the synthesis of Cs_2_AgBiBr_6_, was studied by varying the concentration of each ligand while keeping other reaction parameters fixed. Optimal conditions for obtaining large NSs were observed at concentrations of 1.0–1.5 mL for OA and 1.0 mL for OLAm and a final reaction temperature of 250°C. Figure 2b shows the TEM micrographs of Cs_2_AgBiBr_6_ samples prepared at various concentrations of OA. A lower concentration of OA (0.5 mL) largely produces small nanoplatelets of ∼5–80 nm size and some byproducts of AgBr, as seen in the XRD data (Figure S7a). The interaction between OA and the cations on the surface of Cs_2_AgBiBr_6_ nanocrystal is very weak, while OLAm binds strongly to the nanocrystals (NCs), as earlier reported by Zhang et al. in 2019. Zhang et al. backed this with experimental evidence from nuclear magnetic resonance (NMR) and nuclear overhauser effect (NOE) spectroscopy studies [33]; however, the fundamental reason for the weak interaction of OA with the surface of the NCs has not yet been fully clarified. Notwithstanding, since OA and OLAm are both L‐type ligands but with a carboxylic and amine headgroups, respectively [34], the pH of the nanocrystal's surface was probably not basic enough to allow for the deprotonation of OA to form NCs‐OA bonds. An increase in pH has been previously linked to enhanced deprotonation of OA on material's surfaces [35, 36]. Nonetheless, the low amount of the OA ligand limits the acid‐base equilibrium of the binary ligands needed to provide a stable ligand shell for the crystal growth of Cs_2_AgBiBr_6_ NSs. Further increase in OA concentration above 1.5 mL results in the formation of inhomogeneous NSs. The uniformity of the crystals and the formation of byproducts were highly sensitive to the amount of OLAm in the reaction system, since OLAm binds strongly to the surface of the nanocrystals. Figure 2b presents the TEM images of Cs_2_AgBiBr_6_ samples prepared at various concentrations of OLAm. The OLAm concentration of 1.0 mL favours the growth of Cs_2_AgBiBr_6_ NSs with no noticeable byproducts. Low concentration of OLAm led to a limited ligand shell and increased the reactivity in the system, leading to the growth of inhomogeneous sheets. Meanwhile, a further increase in OLAm concentration to 1.5 mL largely produces thick micro‐ and nanocrystals and a few nanosheets. Excess OLAm (2.0–2.5 mL) results in the formation of a densely packed ligand shell around the cations, which makes it difficult for complex structures to form and favours the formation of simpler compounds such as AgBr and Cs_3_Bi_2_Br_9_ as byproducts (Figure S8a). So, while an increase or decrease in the ratio of OA/OLAm leads to the formation of byproducts, OA largely affects the lateral dimension of the nanocrystals, while OLAm significantly influences the uniformity of the NSs and their structural integrity. Interestingly, the excitonic absorption peak and PL emission positions were not affected by the variation of OA (Figure S7b,c) and OLAm (Figure S8b,c) concentrations in the reaction mixture: an indication of a relatively stable band structure that is not affected by morphological changes. Also, the histograms for the lateral size distribution, including the statistical details of the estimated mean size and SD values for nanostructures that were synthesized using different concentrations of OA and OLAm, are presented in Figure S6d–k, respectively, and further discussed in Section SI2.

### 2.3 Photodetection Properties of Cs_2_AgBiBr_6_ Nanosheets

Generally, photodetection involves the absorption of photons by a suitable material followed by optical transitions that produce free charge carriers, which are extracted at the electrodes with the aid of an electric field to yield photoinduced current. Cs_2_AgBiBr_6_ is a promising material for many applications in optoelectronic devices, especially where photon absorption and charge extraction are paramount, such as photodetection. Moreover, Cs_2_AgBiBr_6_ is Pb‐free and has exhibited superior stability under ambient conditions, temperature, and exposure to light over other conventional halide hybrid organic‐inorganic perovskites such as MAPbBX_3_ (X = I, Br, or Cl) [37, 38]. In addition, they have shown desirable properties suitable for photodetection applications, such as a long carrier lifetime [7, 39], a low minority carrier diffusion length of 700 nm up to 2.44 µm (down to 123 K) [3], and high resistivity that results in low noise and reduced dark current [40].

**FIGURE 3:**
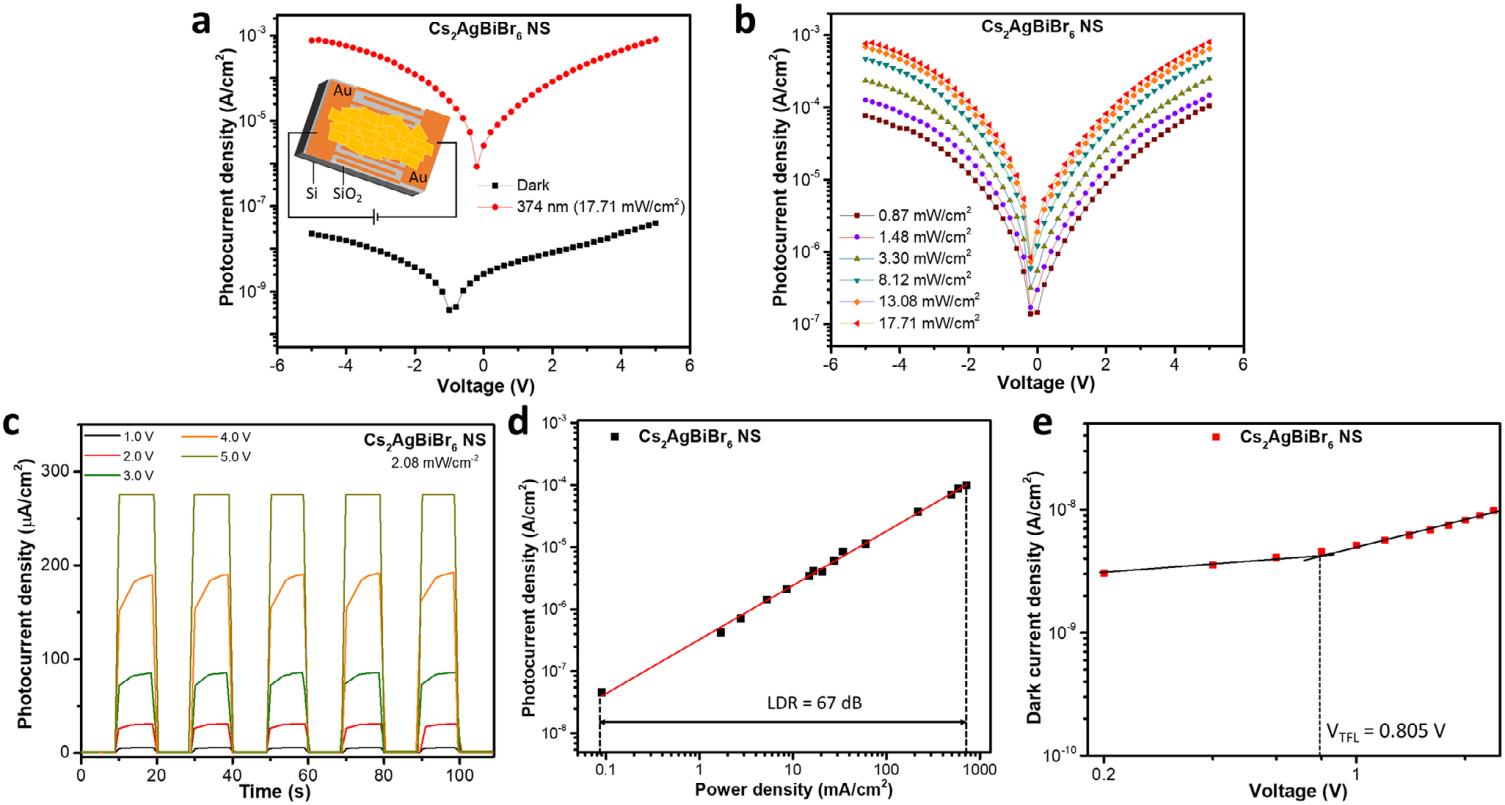
Current–voltage characteristics of Cs_2_AgBiBr_6_ NSs photodetector. (a) current under dark and illumination conditions within a voltage bias range of −5.0 to 5.0 V: the inset shows the schematic diagram of the photodetection device, (b) photocurrent at various incident light power densities and different voltages, (c) transient photocurrent response at various voltages and fixed illumination power of 2.08 mW/cm^2^ (pulse frequency of 50 mHz and sampling interval of 1 s), (d) photocurrent density at different power densities obtained at 4.0 V and used to evaluate the LDR, and (e) a plot of dark current density against different voltages. All measurements were performed using the 374 nm laser, except that a 405 nm laser was used to access much higher power densities for the adequate evaluation of the linear dynamic range (LDR) data presented in Figure 3e.

A schematic representation of the fabricated quasi‐2D Cs_2_AgBiBr_6_ NSs‐based photodetection device is presented in the inset of Figure 3a with an expanded view given in Figure S9a. The device consists of the colloidal Cs_2_AgBiBr_6_ NSs deposited onto Si/SiO_2_ substrates with interdigitated Au electrodes, and the SEM micrograph, which shows all the regions on the device's surface, is given in Figure S9b. The device was annealed at 200°C for 10 min to improve ohmic contacts and remove some insulating ligand barriers that could limit charge transport and device performance. The SEM image of the NSs before and after annealing is shown in Figure S9c,d, respectively. The micrographs disclosed Cs_2_AgBiBr_6_ NSs for the annealed device and unannealed sample, with no noticeable difference in shape and size. The UV–vis absorption of the NSs that were annealed and redispersed into toluene also shows an absorption spectrum similar to the freshly prepared colloidal samples and comparable confined excitonic peak positions (Figure S10a). Furthermore, the XRD of the annealed and the unannealed colloidal NSs drop‐casted onto glass substrates shows a similar XRD pattern for both samples (Figure S10b). The microstrain (ε) in the crystal lattice was evaluated from XRD data using the Williamson‐Hall method, and similar values of 1.15 and 1.21 × 10^−3^ were obtained for both annealed and unannealed Cs_2_AgBiBr_6_ NSs, respectively (Figure S11a,b). Therefore, both optical and structural analysis suggest that the annealing of the photodetection device did not significantly alter the structural and optical features of the NSs. Furthermore, Fourier transform infrared (FTIR) spectroscopy measurements performed on annealed and unannealed samples show the partial removal of ligands, as the characteristic peaks associated with OLAm were observed for both samples, as seen in Figure S12. The peaks at 2923 and 2854 cm^−1^ are assigned to the CH_2_ asymmetric and symmetric stretching modes in the OLAm chain, respectively. The peak observed at 1579 cm^−1^ is related to the NH_2_ scissoring vibration and N‐H bending motion, while the band at 1467 is linked to CH_2_ scissoring in OLAm [41, 42]. The characteristic peak for OA observed at 1711 cm^−1^ was not observed in the annealed and unannealed samples. We conclude that annealing the photodetection devices at 200°C for 10 min is not sufficient to remove all the ligands in the samples.

The current density‐voltage curves of the Cs_2_AgBiBr_6_ NSs photodetection device in dark and under illumination conditions are presented in Figure 3a. A dark current density of 4.0 × 10^−8^ A/cm^2^ was observed for the photodetection device at a bias voltage of 5V. This low dark current response is likely due to the charge injection barrier at the metal‐semiconductor interface [43, 44]. The photocurrent notably increases under illumination and produces a remarkable on‐off ratio of 3.04 × 10^3^ and 2.39 × 10^4^ at 0.87 and 17.7 mW/cm^2^, respectively. The on/off ratio of the photodetection device increases with increasing power density (Figure S13a) due to enhanced photogeneration of charge carriers. The high on‐off ratio recorded for the photodetection device is attributed to the low dark current attained by the device, coupled with the high quality of the NSs, and the reduction of detrimental trap states at grain boundaries because of their large lateral sizes and long charge carrier lifetimes, which are beneficial for charge extraction and high device performance. The large lateral dimension of the synthesized NSs will bridge the electrode spacing of the device more efficiently, and limit defects due to grain boundaries and the consequent charge recombination to improve charge transport and photodetection performance [28]. More so, unlike many Pb‐based organic‐inorganic perovskites such as MAPbX_3_, the Cs_2_AgBiBr_6_ NSs could withstand high‐temperature calcination (200°C) without any significant distortion of their crystal structure, which was vital in improving charge transport and device performance. A positive zero‐point deviation of 0.79 V was observed for the photocurrent relative to dark current (Figure 3a). Halide perovskites inherently experience high ionic mobility along with electronic contribution to charge transport, and the movement of ions can influence internal electric fields and induce zero‐point drifts in their *I*–*V* characteristics. Under illumination, the ions would acquire more energy and attain enhanced migration and accumulation at the interfaces. So, the zero‐point deviation observed for photocurrent relative to the dark current is attributed to pronounced ion migration under illumination [45].

The dependence of photocurrent on incident power density is presented in Figure 3b and Figure S13b. The photocurrent increases with increasing power density of the incident photons because of the enhancement in the photogeneration of excitons. The photodetection device mildly treated at 50°C for 30 min produces a photocurrent that is only 10^3^ higher than the dark current at 17.7 mW/cm^2^ (Figure S14a,b): one order less than the value obtained for the device annealed at 200°C (Figure 3a). The reduced performance is attributed to poor ohmic contact at the Au‐Cs_2_AgBiBr_6_ NSs interface, which would increase the charge injection barrier at the interfaces. In addition, the mildly treated devices (at 50°C) are expected to contain more non‐conducting ligands compared to devices annealed at 200°C because of the increased calcination temperature, which may further limit their photodetection performance (Figure S12). However, since the ligands were not completely removed after annealing at 200°C, the contribution of the partial removal of ligands toward improved charge transport may be limited. The dark current observed for the devices annealed at 200°C increased by 3.34 times (at 5.0 V) more than the values obtained for the devices treated at 50°C. This further affirms reduced resistance to charge transport in 200°C‐treated devices (Figure S14c). The annealing of Cs_2_AgBiBr_6_‐based photodetectors at 75–285°C has been previously reported, owing to the good thermal stability of Cs_2_AgBiBr_6_ [15, 46, 47]. Transient photocurrent density was also measured at different bias voltages for the Cs_2_AgBiBr_6_ NSs photodetection device at an incident power density of 2.08 mW/cm^2^ and a signal frequency of 50 mHz (equivalent to a laser on/off duration of 20 s). The photocurrent density increases with increasing bias voltage (Figure 3c) due to the enhancement of the electric field and carrier drift velocity that promote charge transport and extraction. In a similar measurement at a constant voltage of 2.0 V, the transient photocurrent also increases with increasing power density of the incident photons as expected (Figure S15a). A linear dynamic range (LDR) was deduced for the Cs_2_AgBiBr_6_ NSs photodetectors from their photocurrent density measurements at various power densities, as given in Figure 3d. The LDR was evaluated using Equation 1 [21]:

(1) $$LDR=20\log\left(\frac{I_{\max}}{I_{\min}}\right)$$

where I_max_ and I_min_ denote the maximum and minimum photocurrent within the linear range, respectively. The LDR evaluated for the device is 67 dB. Lower LDR values have been reported for some Cs_2_AgBiBr_6_‐based photodetectors [48, 49], as well as many transport‐layer‐free Pb‐based organic‐inorganic hybrid photodetectors [50, 51]. The LDR defines the region within which the output of the photodetection device scales linearly with respect to the intensity of the incident light input: a necessary condition for sustaining a consistent responsivity [52]. The plot of photocurrent against incident power was fitted to the power law I = AP^θ^, where A is a constant, and θ is a parameter that reflects the trapping and recombination processes of photogenerated carriers in the device (θ < 1) [53]. After fitting the plot to the above relation, a θ value of 0.69 was obtained for the photodetection device (Figure S15b). Fractional values of θ close to 1 are desirable as they indicate suppression of charge recombination processes in the device [43]. The θ value obtained for the Cs_2_AgBiBr_6_ NSs photodetector heated at 50°C was 29.0% less than the value deduced for the device annealed at 200°C during device fabrication (Figure S15b,c).

Furthermore, to evaluate the trap density, N_t_, of the Cs_2_AgBiBr_6_ NSs photodetector, the space charge limited current (SCLC) analysis was performed, and the dark current density is plotted against various bias voltages to obtain the value of the trap filling limit (TFL) voltage, V_TFL_, as shown in Figure 3e. A linear response is seen in the ohmic region at lower voltages, as indicated in the plot. At a higher voltage, a deviation from this ohmic behavior is observed at the trap filling limit, where a V_TFL_ value of 0.805 V was obtained for the device. At the TFL, the trap states are occupied by charge carriers. So, the trap density can be evaluated using Equation 2:

(2) $$N_{t}=\frac{2\varepsilon_{r}\varepsilon_{o}V_{TFL}}{eL^{2}}$$

where the relative dielectric constant is represented by *ɛ_r_* = 51 [29], *ɛ_o_* denotes the vacuum permittivity, *e* is the electronic charge, and the channel length, *L* = 5 µm. The N_t_ of 1.82 × 10^14^ cm^−3^ was evaluated for the photodetection device. Limiting trap states is paramount for reducing charge recombination rates and improving carrier extraction to boost photodetection performance. The trap density calculated for the quasi‐2D Cs_2_AgBiBr_6_ NSs is relatively high compared to some reported values for its 3D counterparts [17]. However, the *N_t_* value obtained for NSs is still within reasonable limits for double perovskite materials [54]. A few factors that could have influenced the *Nt* value evaluated for the NSs‐based photodetectors are proposed. First, the NSs prepared are quasi‐2D nanostructures and will generally have more surface defects due to confinement and a high surface‐to‐volume ratio when compared to their 3D counterparts. Also, the photodetectors prepared consist of a network of NSs, and defects due to grain boundaries will also contribute to the *N_t_* value. Reports on the *N_t_* values of 2D/quasi‐2D Cs_2_AgBiBr_6_‐based photodetectors are scarce in the literature. Nonetheless, the trap density calculated for the photodetectors is within the range of some reported values for the material. While lower trap density in the order of 10^12^ cm^−3^ has been reported for spin‐coated Cs_2_AgBiBr_6_ [17], much higher values in the order of 10^16^ cm^−3^ have been reported for some solution‐processed films of Cs_2_AgBiBr_6_ [54].

**FIGURE 4:**
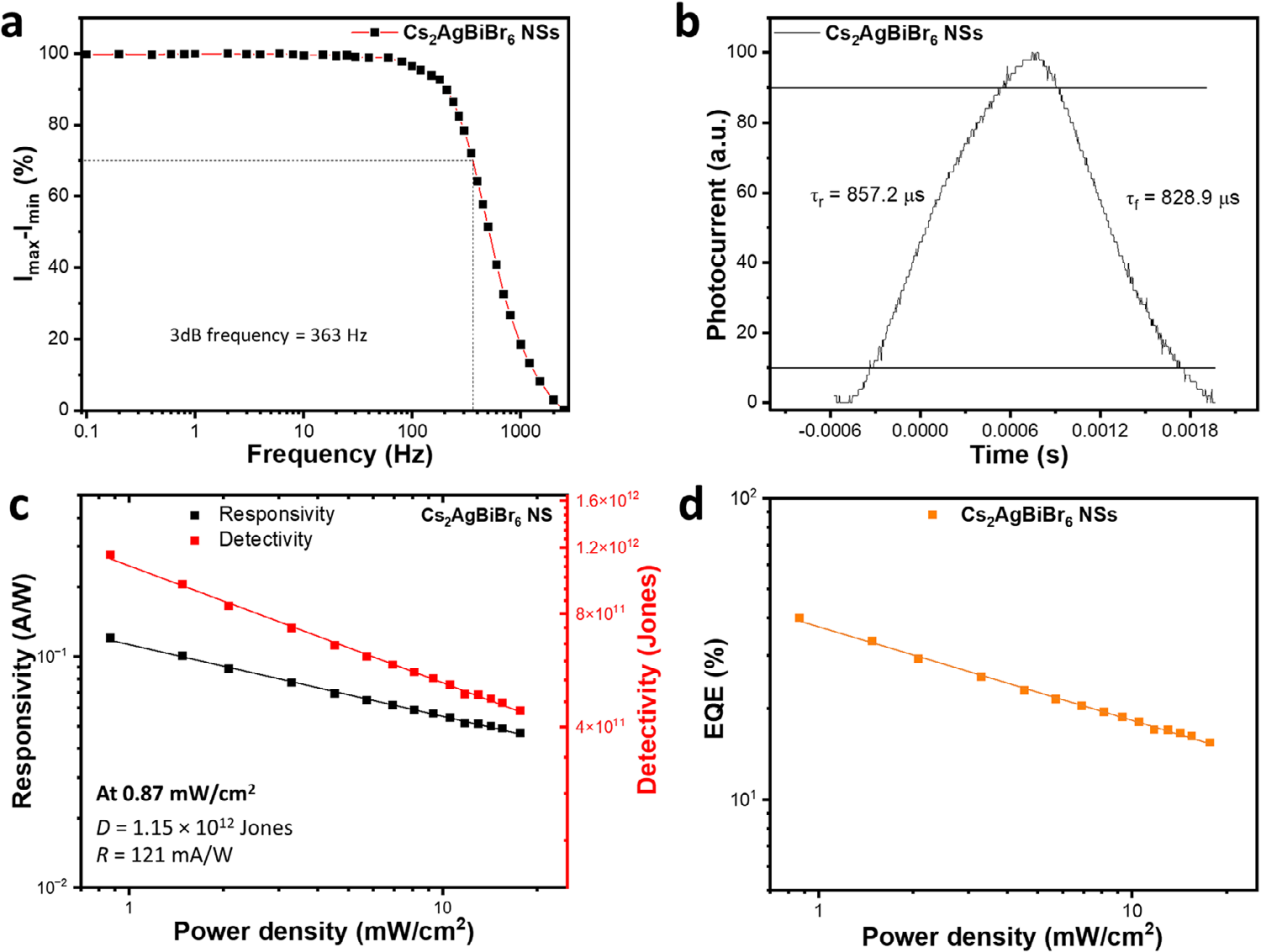
Cs_2_AgBiBr_6_ NSs photodetector. (a) frequency responses obtained under 4.0 V bias voltage and 2.08 mW/cm^2^ illumination pulse power, (b) rise and fall times at 400 Hz, (c) responsivity, detectivity at different power densities, and (d) external quantum efficiency (EQE), evaluated at 5.0 V bias. All measurements were performed using the 374 nm laser source.

The 3‐dB bandwidth or cut‐off frequency, which characterizes the device's speed, was obtained from the frequency responses measured at laser pulsing frequencies of 0.05 to 2500 Hz. The normalized relative photocurrent (I_max_−I_min_) obtained at different frequencies was plotted as shown in Figure 4a and used to deduce the 3dB frequency of the photodetectors at (I_max_−I_min_)/√2, which equates to 70.7% of the maximum I_max_−I_min_ value. A 3dB bandwidth of 363 Hz was obtained for the device. The response times of the Cs_2_AgBiBr_6_ NSs‐based photodetectors were evaluated at 400 Hz (near the 3dB frequency) and yield fast rise and fall times of 857 and 829 µs, respectively (Figure 4b). Despite the intrinsically long lifetimes of the charge carriers, these response times outperformed many pristine and modified Cs_2_AgBiBr_6_‐based photodetectors and some conventional organic‐inorganic Pb‐based perovskite photodetection devices, as outlined in Table 1. The fast response times obtained for the device can be attributed to the good quality of colloidal quasi‐2D nanocrystals and the large lateral size of the NSs that effectively bridged the electrode spacing and limited traps due to grain boundaries, which is beneficial for efficient charge transport.

**TABLE 1:** Photodetection properties of pristine Cs_2_AgBiBr_6_ and Cs_2_AgBiBr_6_‐based devices that consist of heterostructures and/or engaged transport layers in their architectures, and conventional organic‐inorganic halide Pb‐based perovskite photodetectors.

Type of perovskites	Material	Voltage (V), source wavelength (nm)	D (Jones)	R (A/W)	Rise/fall time (ms)	Refs.
Pristine Cs_2_AgBiBr_6_	Cs_2_AgBiBr_6_	5.0, 375	1.15 × 10^12^	0.12	0.857 / 0.829	This work
	Cs_2_AgBiBr_6_	0.0, white light,	2.5 × 10^7^	0.0001	1.3/9.0	[53]
	Cs_2_AgBiBr_6_	5.0, 400	1.38 × 10^9^	0.09	159/85	[56]
	Cs_2_AgBiBr_6_	5.0, 530	4.63 × 10^12^	12.1	110/160	[17]
	Cs_2_AgBiBr_6_	5.0, 520	5.66 × 10^11^	7.01	0.955/0.995	[16]
	Cs_2_AgBiBr_6_	6.0, 530	1.60 × 10^13^	15	∼770	[57]
	Cs_2_AgBiBr_6_	−5.0, 530	1.1 × 10^9^	0.00096	192/271	[43]
Cs_2_AgBiBr_6_‐based (heterostructures and/or engaged transport layers)	TiO2/Cs_2_AgBiBr_6_	0.0, AM 1.5G	5.1 × 10^11^	0.05	160/100	[15]
	CsAgBr_2_/Cs_2_AgBiBr_6_	0.5, 440	4.6 × 10^10^	0.06	1.4 /1.6	[58]
	Cs_2_AgBiBr_6_/ SnO_2_	0.0, 350	2.1 × 10^10^	0.11	< 3 × 10^3^	[40]
	NiO:Mg/Cs_2_AgBiBr_6_/C_60_/BCP	0.0, ‐	4.7 × 10^12^	0.055	382/120 (465 nm laser)	[59]
	PEDOT:PSS/TiO_2_/Cs_2_AgBiBr_6_	−0.3, 405	9.29 × 10^12^	0.666	166/163	[46]
	Cs_2_AgBiBr_6_/TiO_2_	0.0, 405	3.3 × 10^11^	—	2.2/2.7	[60]
Organic–inorganic	MAPbBr_3_‐PdSe_2_	2.0, 520,	5.2 × 10^11^	0.028	0.024/0.025	[61]
	Ni(OH)_2_·0.75H_2_O‐MAPbBr_3_	‐, 365	2.05 × 10^10^	0.0019	84/76 (532 nm laser)	[62]
	MAPbBrCl_2_	0.6, 440	1.41 × 10^11^	0.051	340/920	[50]
	MAPbX_3_(X = Br, l)	0.0, 473 nm	7.01 × 10^11^	0.26	80/580	[63]
	MAPbBr_3_‐TiO_2_	Solar Simulator	∼10^12^	85	90/110	[64]

The photodetection performance of the Cs_2_AgBiBr_6_ NSs was further assessed by evaluating their responsivity (*R*), detectivity (*D*), and external quantum efficiency (*EQE*) using the relations in Equations (3), (4), and 5, respectively [21]:

(3) $$R=\frac{I_{photo}\;-\;I_{dark}}{PA}$$

(4) $$D=\frac{R}{\sqrt{2e\left(I_{dark}/A\right)}}$$

(5) $$EQE=\frac{Rhc}{e\lambda }$$

where *I_photo_* and *I_dark_* are current under illumination and dark conditions, respectively, while *P* denotes the power density of incident light, *A* represents the active area of the photodetector, and *e*, *h*, *c*, and *λ* denote the electron charge, Planck constant, speed of light, and the wavelength of the laser, respectively. The noise equivalent power (NEP) was calculated on the basis that the short noise (*i_s,n_*) (which depends on dark current) is the major source of noise in the device, and other forms of noise, such as thermal and flicker noise, had a negligible impact on the device. Under these conditions, the detectivity is expressed as given in Equation 4, as shown in Section SI3. The responsivity and detectivity of the device were evaluated at 5.0 V, and at different power densities (Figure 4c), while the *EQE* plot is presented in Figure 4d. The responsivity, detectivity, and *EQE* were observed to decrease with increasing laser power density. This behavior is similar to other observations [21, 55] and is associated with the prevalence of bimolecular recombination of charge carriers at higher power [55]. The NSs yield a high detectivity value of 1.15 × 10^12^ Jones, outperforming many reported values for pristine and doped Cs_2_AgBiBr_6_ photodetectors, devices that engage heterojunctions and charge transport layers in their architecture, and conventional organic–inorganic halide perovskites (Table 1). The detectivity of the photodetector defines its capacity to detect weak optical signals and is a vital parameter in determining device performance. The responsivity, which quantifies the photocurrent per unit area for a given optical power, was evaluated and yields 121 mA/W. An *EQE* of 40% was deduced for the device, which represents the ratio of the number of photoexcited charge carriers extracted to produce photocurrent to the number of incident photons.

**FIGURE 5:**
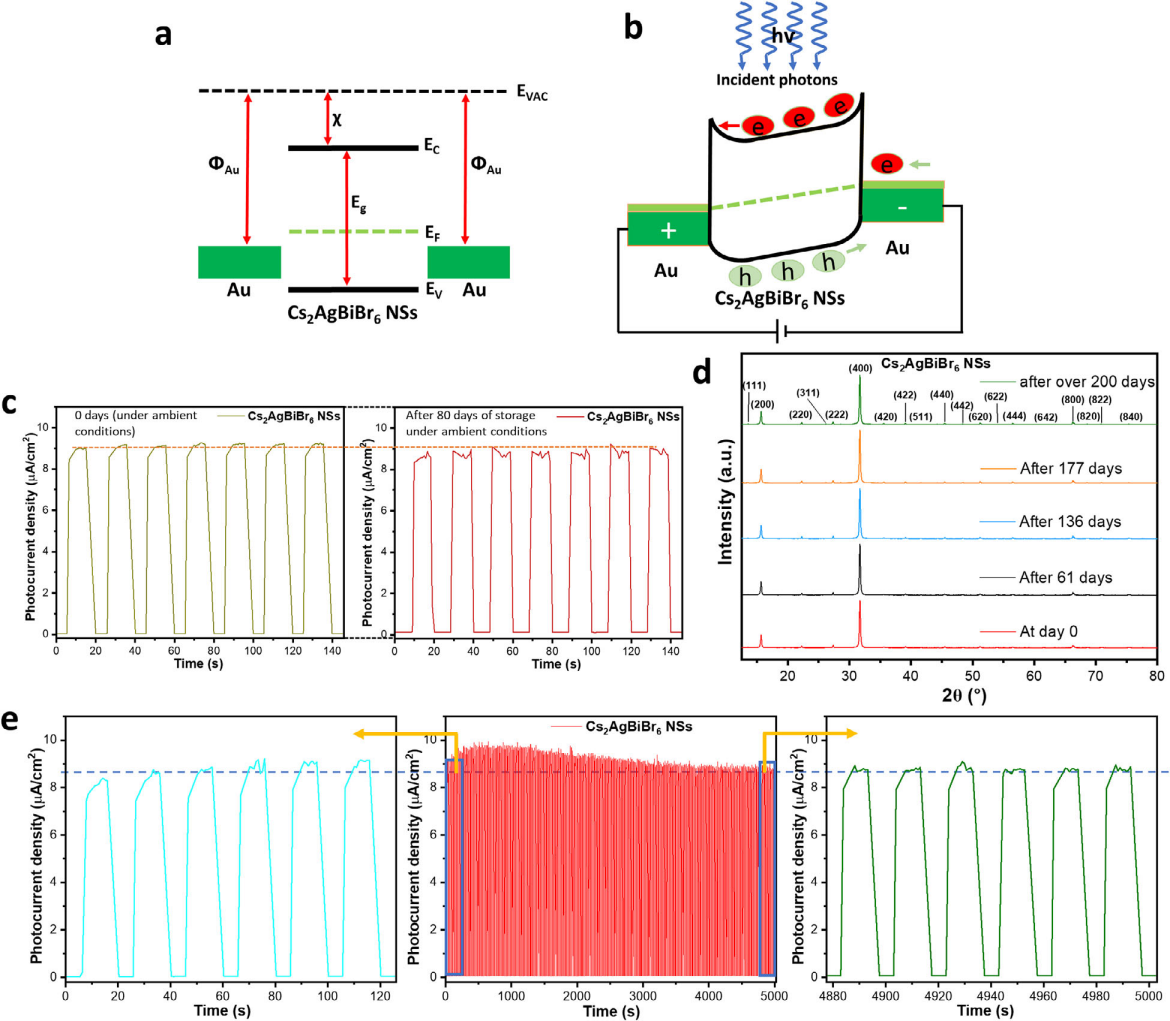
The energy band positions of Cs_2_AgBiBr_6_ NSs and Au electrodes (a) before contact in their equilibrium states and (b) after contact and illumination with incident photons, (c) transient photocurrent of Cs_2_AgBiBr_6_ NSs photodetector at 0.87 mW/cm^2^ illumination power and 3.0 V obtained under ambient conditions at day 0 (initial day) and after storage for 80 days, and (d) XRD patterns of the Cs_2_AgBiBr_6_ NSs between day 0 and after over 200 days of storage in ambient conditions, and (e) the long‐term time‐resolved photocurrent analysis of the photodetection device.

The energy band positions and alignment of the Cs_2_AgBiBr_6_ NSs and the Au electrodes in their equilibrium states before contact are given in Figure 5a. When in contact, electrons will flow from the semiconductor to the metal to attain equilibrium and a common Fermi level, since the work function of Au (5.1 eV) [44] is higher than that of Cs_2_AgBiBr_6_, based on values given in different reports [43, 47, 56]. This will cause energy band bending at both ends of the Au‐Cs_2_AgBiBr_6_ interfaces, as illustrated in Figure 5b. Under dark conditions, charge flow is limited by the energy barrier at the Au‐Cs_2_AgBiBr_6_ interfaces, which contributes to the low dark current observed for the photodetector. Under illumination, photogenerated charge carriers are excited to the conduction band (CB) to form electron‐hole pairs. The electrons in the CB are driven toward the anode while the holes move in the opposite direction. The electric field that results from the external bias will aid the flow of charge carriers and boost the photocurrent generated by the device. Furthermore, since the photodetector is made of stacked layers of interconnected nanosheets, at room temperature, the charge carriers will move from one sheet to the other through both hopping and tunneling mechanisms, and get extracted at the electrodes. The grain boundaries would create defects that serve as trap centers for charge carriers, which will limit charge transport [28]. Hence, the large lateral size observed for the NSs helps to limit grain boundaries and contributes to the high photodetection performance recorded in the presented structures.

The stability of the Cs_2_AgBiBr_6_ NSs photodetectors was investigated under ambient conditions. Transient photocurrent was recorded for the device and repeated after storing the sample under ambient conditions. The device demonstrated excellent stability, showing no noticeable difference in the on/off photocurrent after being kept under ambient conditions for 80 days (Figure 5c). Meanwhile, Pb‐based organic‐inorganic perovskite, MAPbI_3_, photodetector, has been shown to lose 16% of its photocurrent after storage in air for 30 days [63]. The stability of the NSs is further affirmed by the structural stability of the material when kept under ambient conditions for over 200 days. The XRD patterns obtained from intermittent measurements performed on the NSs between day 0 and after over 200 days solely revealed peaks for the cubic phase of Cs_2_AgBiBr_6_ double perovskites, with similar intensities, and no additional peaks for AgBr or other impurities (Figure 5d). Furthermore, the Cs_2_AgBiBr_6_ NSs photodetection device exhibited great long‐term photostability. The initial photocurrent observed in a transient current measurement was sustained after 5000 s (Figure 5e). However, a slight increase in the photocurrent was observed within the initial 550 s before slightly reducing to become consistent after 3300s. A previous work has reported a similar observation in Cs_2_AgBiBr_6_‐based photodetectors and proposed a photo‐activation phenomenon, whereby high‐energy photons induce the healing of defects, as the main reason for this behavior [16]. However, other reasons that could be associated with such behavior, such as the van der Waals interactions between the stacked layers of NSs and the interlayer coupling effects [65, 66]. Also, light‐induced carrier extraction improvement has been observed in other systems, such as TiO_2_/Au nanocrystals, and is related to the work‐function changes after charge carrier accumulation, which changes the material's work function and band alignment. However, this effect is observed during the illumination and not in the long‐term on‐off experiments, as in our case [67]. While this observation opens an interesting perspective regarding the photostability evaluation of Cs_2_AgBiBr_6_ NSs photodetectors, further investigation will be required to adequately explain such behavior.

## 3 Conclusions

In summary, in this work, quasi‐2D Cs_2_AgBiBr_6_ nanosheets (NSs) were developed using the low‐temperature injection colloidal synthesis and used to fabricate high‐performance photodetectors. The reaction temperature and the concentration of ligands (OA and OLAm) played significant roles in the shape, size, and structural integrity of the synthesized Cs_2_AgBiBr_6_ NSs. The tuning and optimization of the ligands in the reaction system allowed for the synthesis of Cs_2_AgBiBr_6_ NSs at a higher final reaction temperature (250°C), which leads to the formation of larger sheets, without any structural distortion. Cs_2_AgBiBr_6_ NSs of up to 1.4 µm lateral sizes, which are only a few nm thick, were synthesized. The high‐performance photodetectors fabricated using the Cs_2_AgBiBr_6_ NSs yielded a notably high detectivity and on‐off ratio of 1.15 × 10^12^ Jones and 2.39 × 10^4^, respectively. The device also recorded responsivity of 121 mA/W, and a fast sub‐millisecond rise and decay time of 857 and 829 µs, respectively. The performance of the Cs_2_AgBiBr_6_ NSs photodetector is remarkable, especially because the device is free of transport layers, which often complicates device design and reduces the cost‐effectiveness for practical applications. This work demonstrates the potential of micro‐sized colloidal quasi‐2D Cs_2_AgBiBr_6_ lead‐free double perovskite NSs with desirable characteristics for the design of high‐performance photodetectors and other optoelectronic applications.

## 4 Experimental

### 4.1 Chemicals

Silver nitrate (AgNO_3_, 99.8%), bismuth bromide (BiBr_3_, >98.0%), Cesium carbonate (Cs_2_CO_3_, 99.9%), 1‐octadecene (ODE, technical grade 90%), oleic acid (OA, technical grade 90%), oleylamine (OLAm, technical grade 70%), hydrobromic acid (HBr, 47%), and toluene (from VWR, 99.5%). All chemicals were purchased from Sigma–Aldrich unless stated, and used as received without further purification.

### 4.2 Preparation of Cs‐Oleate Solution

Ten mL of OLAm was added to 825 mg of Cs_2_CO_3_, which was measured and loaded into a 50 mL 3‐neck flask. In a Schlenk line, under continuous Ar flow, the mixture was heated to 120°C and degassed by applying vacuum pressure for 30 min. The solution was further heated to 150°C, kept at the same temperature for 1 h under Ar flow, and allowed to naturally cool down to room temperature for subsequent use.

### 4.3 Synthesis of Cs_2_AgBiBr_6_

The procedure developed by Liu et al. for the preparation of Cs_2_AgBiX_6_ (X = Cl, Br, and I), nanoplatelets was used with some modifications to the final reaction temperature and the amount of OA used to prepare quasi‐2D Cs_2_AgBiBr_6_ nanosheets. In brief, 0.2 mmol AgNO_3_, 0.1 mmol BiBr_3_, 4 mL ODE, 1.5 mL OA, 1.0 mL OLAm, and 0.1 mL HBr were loaded into a 25 mL three‐neck flask, heated to 120°C under argon flow, and degassed by applying vacuum for 30 min to remove residual air and water. After degassing, the mixture was heated to 200°C for 10 min under argon flow to fully dissolve all precursors and was allowed to cool naturally to room temperature (25°C). At room temperature, 0.3 mL of the prepared Cs‐oleate solution was injected into the mixture and kept for 10 min at room temperature while stirring. The mixture was heated to 250°C and kept for 10 min at this temperature. Afterward, the heating mantle was removed to quench the reaction and cooled to room temperature. The crude solution obtained was centrifuged at 8000 rpm for 5 min to precipitate the sample from the final reaction mixture. The sample was dispersed in toluene and centrifuged again under the same conditions as the crude solution. Lastly, the supernatant was decanted, and the Cs_2_AgBiBr_6_ NSs sample was re‐dispersed in 7.5 mL toluene and stored for further use.

Several samples were also synthesized to investigate the role of temperature and ligands in the colloidal synthesis of Cs_2_AgBiBr_6_ double perovskites. Samples were prepared, following the same procedure described for the NSs synthesis above, but at final reaction temperatures of 210°C, 230°C, 260°C, 270°C, and 290°C. Also, following the same procedure, additional samples were prepared at 250°C and final reaction times of 30 and 60 mins. Furthermore, the role of OA in the reaction was investigated. Additional samples were prepared using 0.5, 1.0, 2.0, and 2.5 mL of OA in the reaction system while maintaining 1.0 mL OLAm and other reaction parameters described for the NSs synthesis above. In another set of samples, the amount of OLAm in the reaction mixture was 0.5, 1.5, 2.0, and 2.5 mL, while 1.5 mL of OA and other parameters were maintained. All samples were cleaned following the same procedure described for the Cs_2_AgBiBr_6_ NSs samples and dispersed in toluene.

### 4.4 Device Fabrication

The Si/SiO_2_ substrates were obtained from Addison Engineering, Inc. San Jose, CA (300 nm ± 5% thermal oxide and resistivity of.005–.020 Ω‐cm) with interdigitated gold electrodes and 5 µm electrode spacing was used for the fabrication of the photodetectors. The SiO_2_ layer provided electrical insulation against current leakage from the Si layer of the substrate. The substrates were cleaned by sonicating in acetone for 2 min and dried with flowing nitrogen gas. 50 µL of the stored Cs_2_AgBiBr_6_ NSs sample was further dissolved in 50 µL of toluene. 20 µL of the resulting solution was drop‐casted onto the Si/SiO_2_ substrate (with interdigitated gold electrodes) and allowed to dry in air. This was repeated once to obtain thin films of Cs_2_AgBiBr_6_ NSs on the substrates. The devices were placed in a vacuum oven and purged twice with Ar to remove any residual air in the chamber. The temperature in the oven was increased to 200°C at a heating rate of ∼2°C/min under flowing Ar gas, calcined for 10 min, and allowed to cool naturally to room temperature to obtain quasi‐2D Cs_2_AgBiBr_6_ NSs‐based photodetection devices.

### 4.5 Photodetection Measurements

The fabricated devices were placed in a probe station and kept under vacuum for evaluation of photodetection properties. The *I*–*V* characteristics of the Cs_2_AgBiBr_6_ NSs photodetection devices were studied by employing the Keithley 4200 semiconductor characterization equipment, with measurements conducted under dark and various incident light power density and voltage bias conditions. The 374 nm excitation laser served as the main incident light source. A 405 nm excitation laser was used to measure *I*–*V* responses under different illumination power densities and bias voltages to study the region within which the photocurrent scales linearly with respect to the intensity of the incident light (the laser provides much higher power density). Transient current responses were also measured for the photodetection device under various illumination power densities and bias voltages. The RIGOL function generator (DG4062) and the illumination for the laser (374 nm), which was converted into a pulsed light, were used in evaluating the device's frequency/time response. Using a power density of 2.08 mW/cm^2^ from the pulse laser, a Tektronix oscilloscope (TDS2014B), and a bias voltage of 4 V (from SRS70, power source), the current‐frequency responses of the photodetection device were measured. The device's active area is 0.0003632 cm^2^.

### 4.6 Characterizations

The prepared samples were further diluted in toluene before drop‐casting onto transmission electron microscopy (TEM) copper grids and used to conduct TEM measurements using a Talos‐L120C and EM‐912 Omega at 120 kV. High‐resolution TEM (HR‐TEM) was conducted on Cs_2_AgBiBr_6_ NSs at 200 KV in STEM mode using a Jeol JEM‐ARM200F NeoARM (probe‐corrected) instrument.

A Zeiss EVO/MA10 instrument was used to conduct scanning electron microscopy (SEM) measurements on Cs_2_AgBiBr_6_ NSs deposited on bare Si/SiO_2_ and the interdigitated gold electrode substrates.

X‐ray diffraction (XRD) patterns of the prepared samples were measured using a Panalytical Aeris diffractometer with a Bragg‐Brentano geometry and a copper anode with an X‐ray wavelength of 0.154 nm from the Cu‐k𝛼1 line.

An AFM (Park Systems XE‐100) instrument in non‐contact mode (ARROW‐NCR‐20, force constant 𝑘 = 42 Nm−1, resonance frequency at 285 kHz) was used to measure the surface topography of the samples, which were prepared by drop‐casting the diluted samples on Si/SiO_2_ substrates.

The UV–vis absorption spectra of the samples were obtained with a Lambda 1050+ spectrophotometer from PerkinElmer equipped with an integration sphere.

FTIR measurements were performed using a PerkinElmer Spectrum Two spectrometer with an ATR unit (diamond).

A spectrofluorometer FS5 fluorescence spectrometer from Edinburgh Instruments was used to obtain the PL emission and PLE spectra of the samples.

The time‐resolved PL measurements were conducted using a picosecond laser attached to the spectrometer with an excitation wavelength of 375 nm and a repetition rate of 100 kHz. The decay profiles are tail‐fitted with a tri‐exponential decay function.

## Conflicts of Interest

The authors declare no conflict of interest.

## Supporting information




**Supporting File**: smll72785‐sup‐0001‐SuppMat.pdf.

## Data Availability

The data that support the findings of this study are available in the supplementary material of this article.
