# Supplementary material for: Colloidal Quasi‐2D Cs2AgBiBr6 Double Perovskite Nanosheets: Synthesis and Application as High‐Performance Photodetectors
Source: Small. 2026 Feb 9;22(21):e13500. doi: 10.1002/smll.202513500 (PMC13081099; doi:10.1002/smll.202513500)
Supplement: Supplementary file 1 — Supporting File: smll72785‐sup‐0001‐SuppMat.pdf. [file SMLL-22-e13500-s001.pdf]

Supplementary Information

Colloidal quasi-2D $Cs_2AgBiBr_6$ double perovskite nanosheets: synthesis and application as high-performance photodetectors

Pannan I. Kyesmen[1], Eugen Klein[1], Brindhu Malani S[1], Rostyslav Lesyuk[1,2], and Christian Klinke[*1,3]

[1] *Institute of Physics, University of Rostock, Albert-Einstein-Str. 23-24, 18059 Rostock, Germany*

[2] *Pidstryhach Institute for applied problems of mechanics and mathematics of NAS of Ukraine, Naukowa str. 3b, 79060 Lviv, Ukraine*

[3] *Department Life, Light & Matter, University of Rostock, Albert-Einstein-Strasse 25, 18059 Rostock, Germany*

* Correspondence should be addressed: christian.klinke@uni–rostock.de

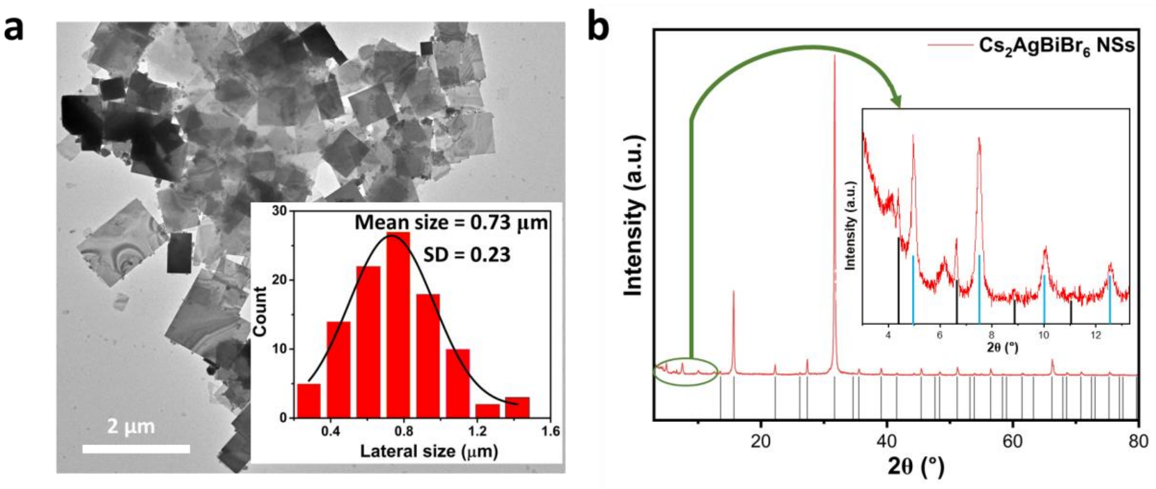

Figure S1. $Cs_2AgBiBr_6$ nanosheets (NSs). a) TEM image at lower magnification: the inset shows the histogram for the lateral size distribution of the NSs, and b) XRD pattern with an expanded view of low-angle periodic diffraction peaks that affirm the stacking of the NSs.

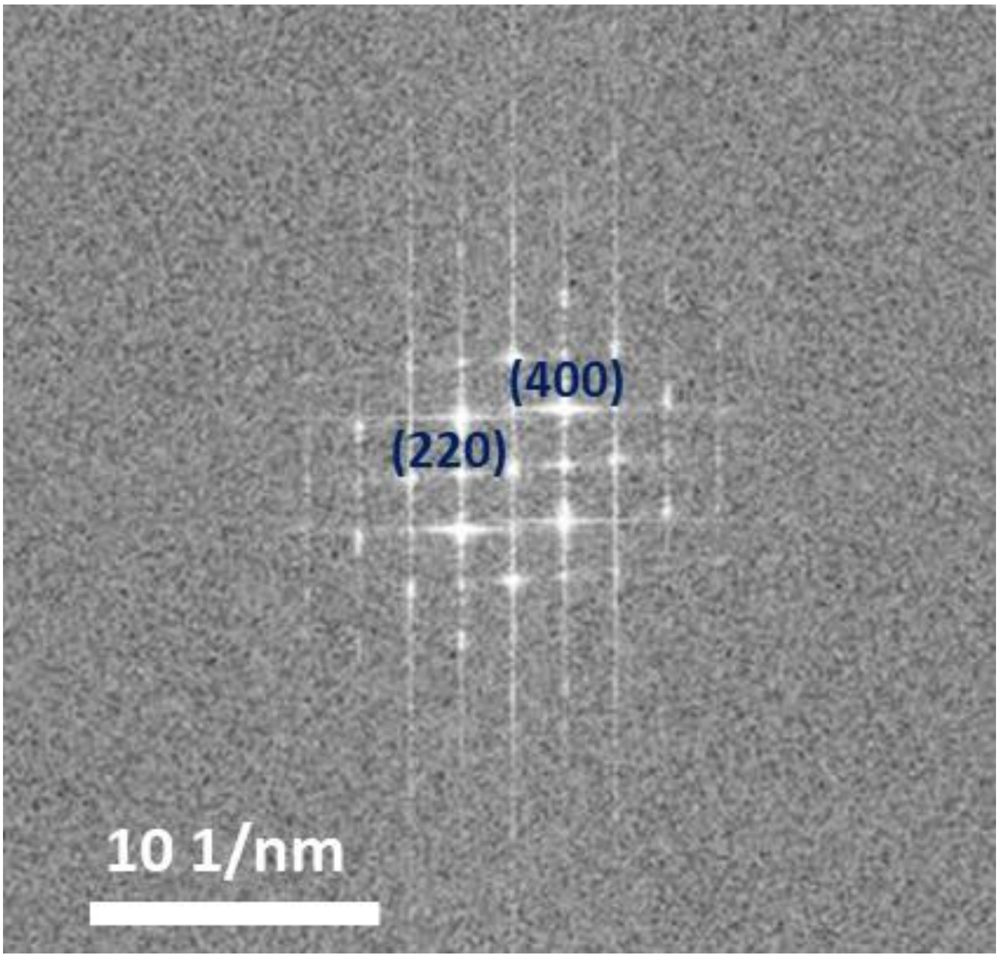

Figure S2. The FTT pattern derived from the HRTEM image of the $Cs_2AgBiBr_6$ NSs.

Table S1. The d-spacing and lattice size as calculated for some selected planes of the XRD data of $Cs_2AgBiBr_6$ NSs using Bragg's Law and the Miller indices, and compared to the d-spacing evaluated from the SAED pattern obtained for the NSs.

2θ (°)	Miller indices (hkl)	d-spacing calculated from XRD data (nm)	Calculated lattice size, a (nm)	d-spacing calculated from SAED image (nm)
15.6929	200	0.564	1.128	0.564
22.1886	220	0.400	1.132	0.397
31.6336	400	0.283	1.130	0.285
35.5319	420	0.252	1.129	0.255
51.1817	620	0.178	1.128	0.179
66.1835	800	0.141	1.128	0.142
68.6063	820	0.137	1.127	0.136

SI1

Bragg's law, $d = n\lambda/2nsin\theta$, and the Miller indices relation that defines lattice size, $a = d\sqrt{h^2 + k^2 + l^2}$ were engaged in calculating lattice distance and size for the $Cs_2AgBiBr_6$ NSs from the XRD data [1]. An approximate lattice size of 1.13 ± 0.03 nm was obtained for the $Cs_2AgBiBr_6$ nanosheets. The estimation of the d-spacing from the SAED pattern in **Figure 1d** was done using the ImageJ software.

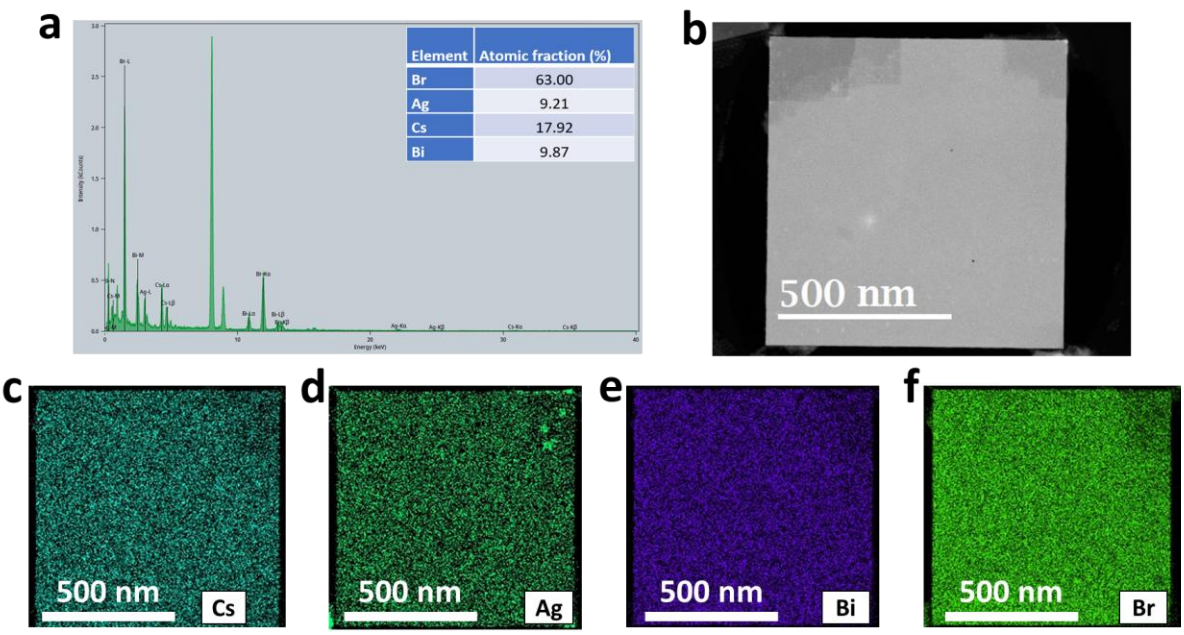

Figure S3. (a) EDS analysis of $Cs_2AgBiBr_6$ NSs with the inset showing atomic fraction for individual elements, b) HRTEM micrograph of $Cs_2AgBiBr_6$ NSs, and c), d), e), and f) show the EDS maps for Cs, Ag, Bi, and Br, respectively.

Table S2. Time constants τ_i and amplitudes A_i (for i = 1, 2, and 3) for the PL decay components of $Cs_2AgBiBr_6$ NSs, deduced after fitting the time-resolved PL to a tri-exponential decay function, $I(t) = A_1 e^{-\frac{t}{\tau_1}} + A_2 e^{-\frac{t}{\tau_2}} + A_3 e^{-\frac{t}{\tau_3}}$. The corresponding fitting errors obtained for the decay components are also included in the table.

Sample	τ_1 (ns)	τ_2 (ns)	τ_3 (ns)	$A_1 \times 10^3$	$A_2 \times 10^3$	$A_3 \times 10^3$
**$Cs_2AgBiBr_6$ NSs**	55.1 ±1.9	316.8 ±5.9	4841.3 ±107.3	68.9 ±10.0	1.25 ±0.04	0.107 ±0.001

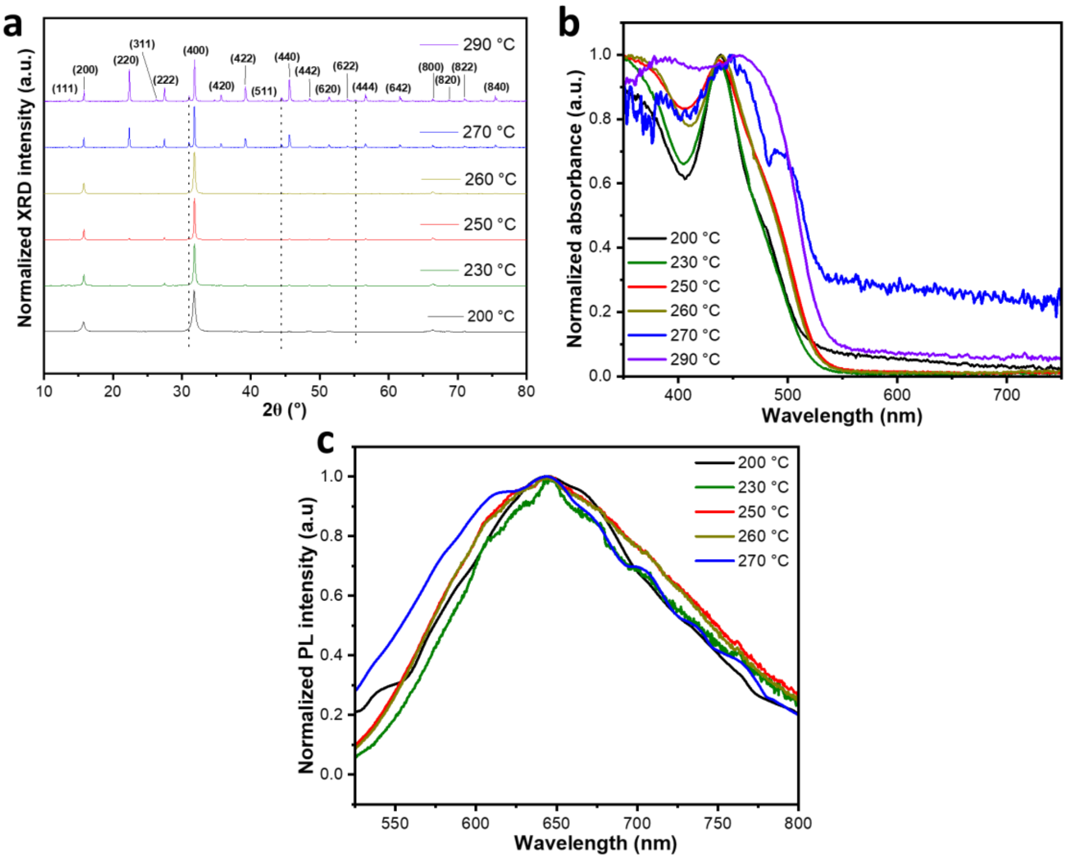

Figure S4. Synthesized $Cs_2AgBiBr_6$ samples prepared at various final reaction temperatures. a) XRD patterns, b) normalized UV-vis absorption spectra, c) normalized PL emission spectra.

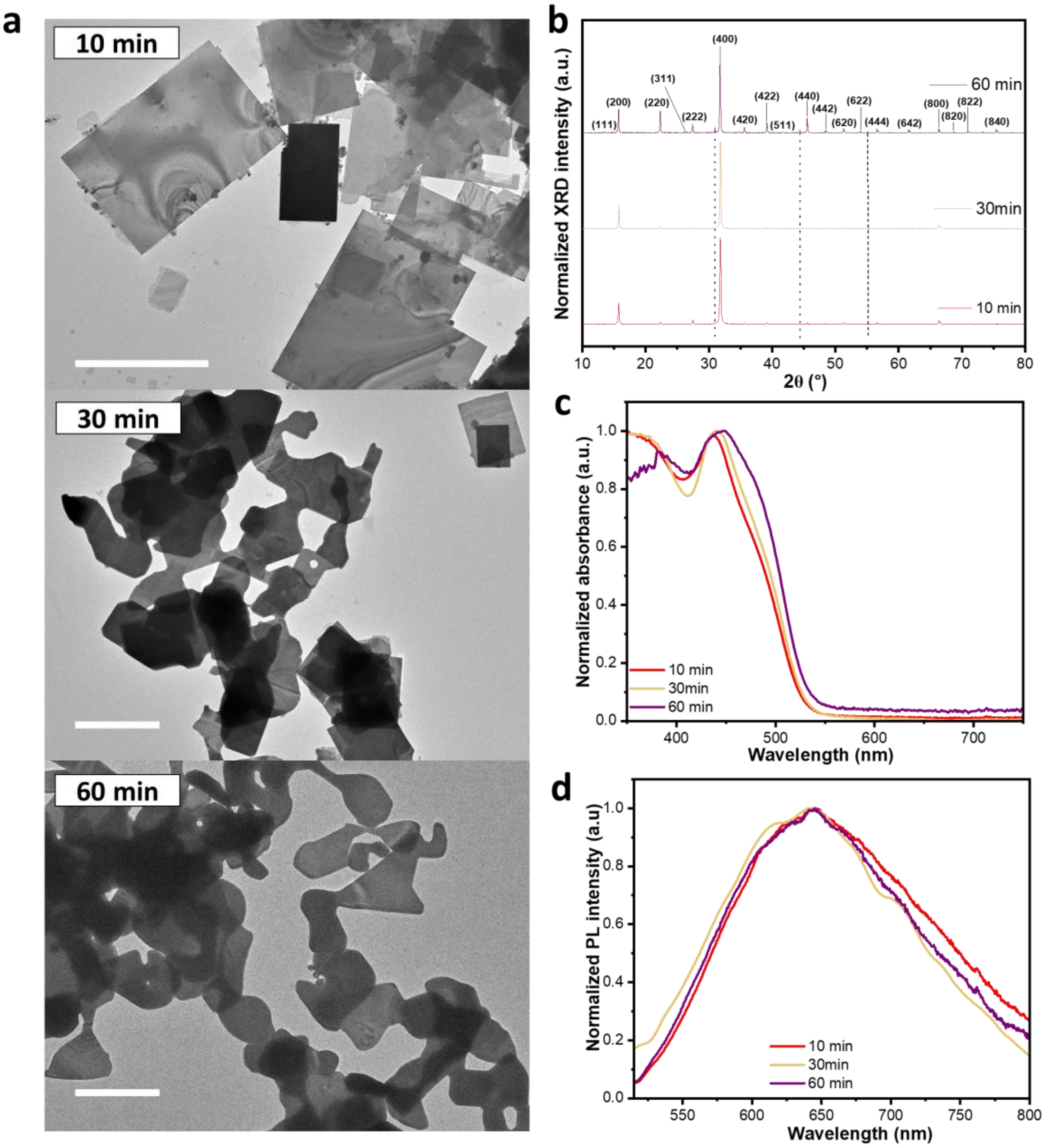

Figure S5. Synthesized $Cs_2AgBiBr_6$ samples prepared at different final reaction temperatures. a) TEM micrographs, b) XRD patterns, c) normalized UV-vis absorption spectra, and d) normalized PL emission spectra. The scale bars shown in the micrographs represent 1 μm.

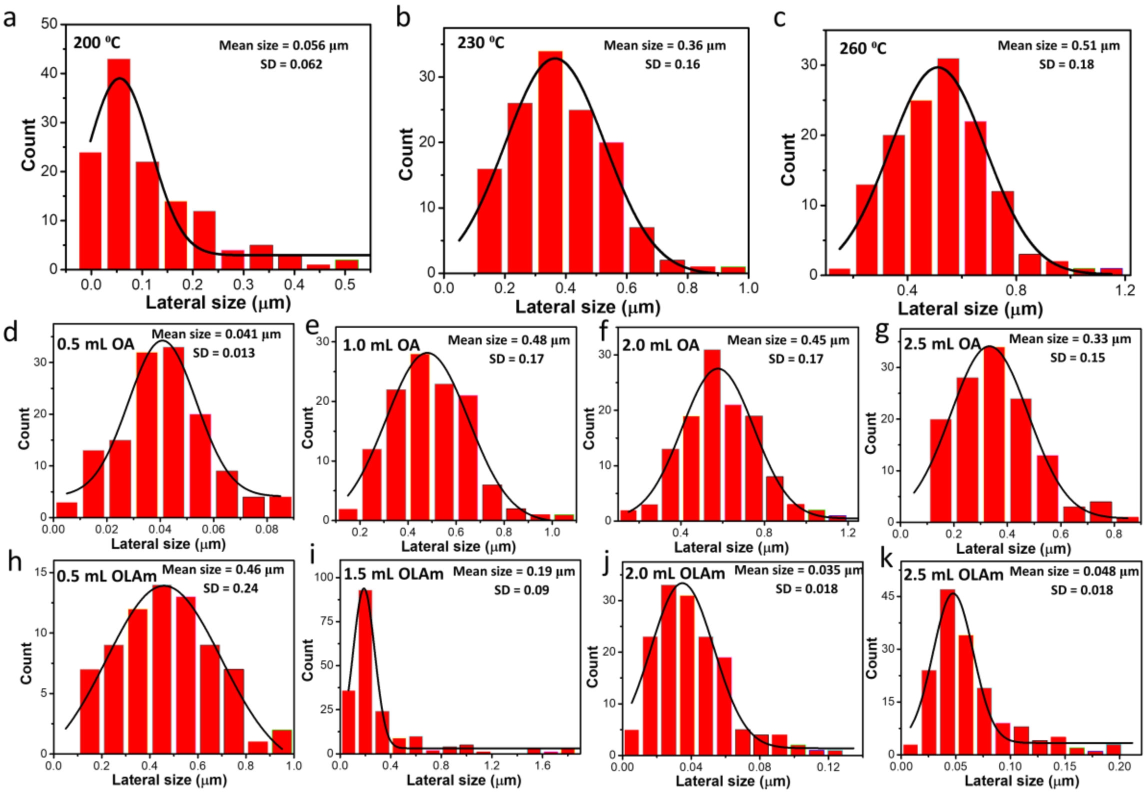

Figure S6. Histograms for the lateral size distribution of $Cs_2AgBiBr_6$ samples prepared (a-c) at different final reaction temperatures, and using various concentrations of (d-g) oleic acid (OA), and (h-k) oleylamine (OLAm), respectively. For clarity, note that the histogram for the lateral size distribution of the samples prepared at the reaction temperature of 250 °C, which contain 1.5 and 1.0 mL of OA and OLAm, respectively, in the reaction mixture, is given in the inset of Figure S1a.

SI2

The lateral size distribution analysis performed on the TEM images of the nanostructures using ImageJ software provides vital statistical clarity on the sizes of the synthesized $Cs_2AgBiBr_6$. The samples prepared at 250 ºC, consisting of 1.5 and 1.0 mL of OA and OLAm in the reaction system, respectively, yielded the maximum mean size of 0.73 µm with a standard deviation (SD) of 0.23 for the NSs (inset of **Figure S1a**). This SD value represents 31.5 % of the mean size and is the minimum value estimated among all the prepared nanostructures. The SD represents the variation of the size around the mean value. Samples prepared at 200 ºC yielded an SD value that is 110.7 % of the mean value, representing the highest variation around the average value recorded for the samples (Figure S6a). Other samples prepared at different temperatures (**Figure S6b** and **S6c**), and using various concentrations of OA (**Figure S6d-g**) and OLAm (**Figure S6d-g**), produced mean size values ranging from 0.035-0.51 µm with SD values that are 31.7-52.2% of their average sizes.

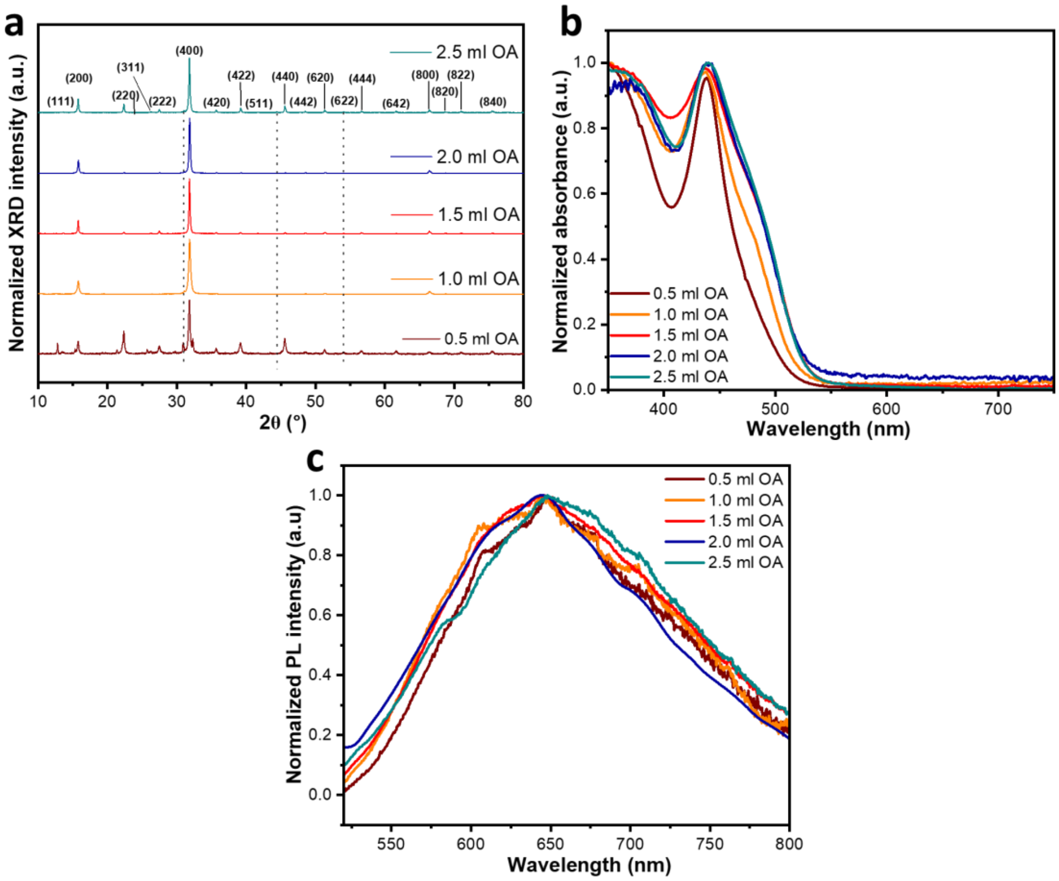

Figure S7. Colloidal $Cs_2AgBiBr_6$ samples that were prepared using different concentrations of Oleic Acid (OA): showing their a) XRD patterns, b) normalized UV-vis absorption spectra, and c) normalized PL emission spectra, respectively.

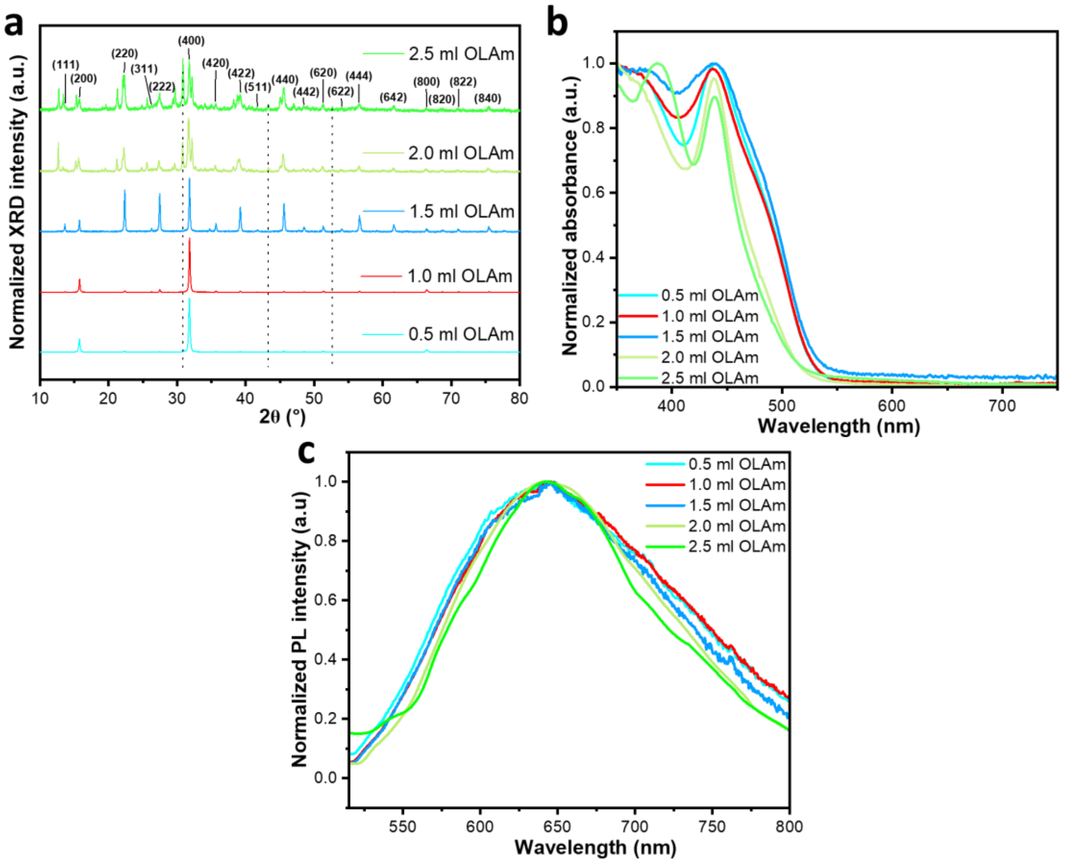

Figure S8. Colloidal $Cs_2AgBiBr_6$ samples that were prepared using different concentrations of Oleylamine (OLAm): showing their a) XRD patterns, b) normalized UV-vis absorption spectra, and c) normalized PL emission spectra, respectively

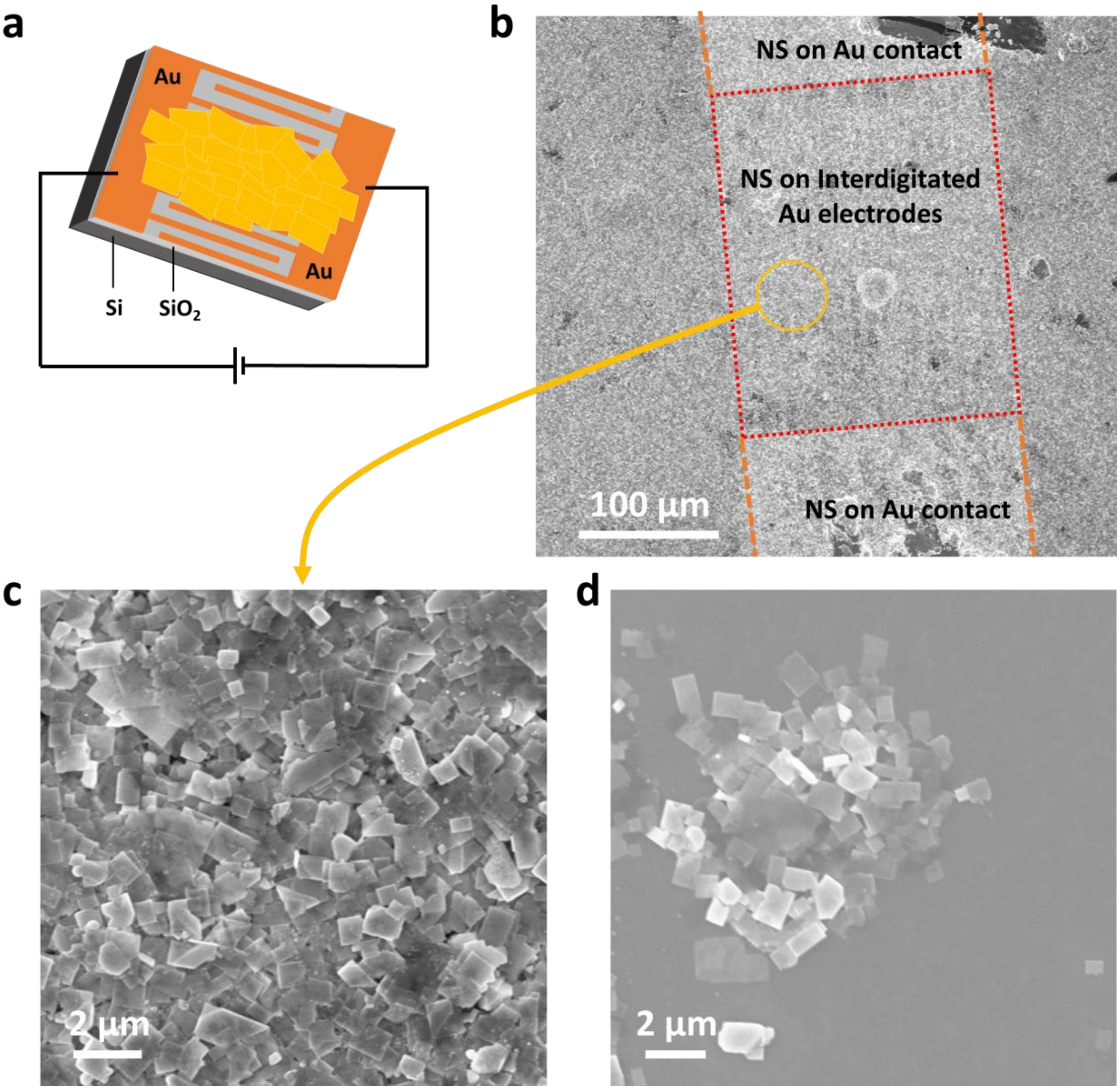

Figure S9. Photodetector based on $Cs_2AgBiBr_6$ NSs. a) schematic diagram of the fabricated device, b) SEM micrograph of the photodetection device, while c) presents an expanded view of the NSs (annealed at 200 °C), and d) shows the SEM image of NSs deposited on Si/SiO_2 substrates.

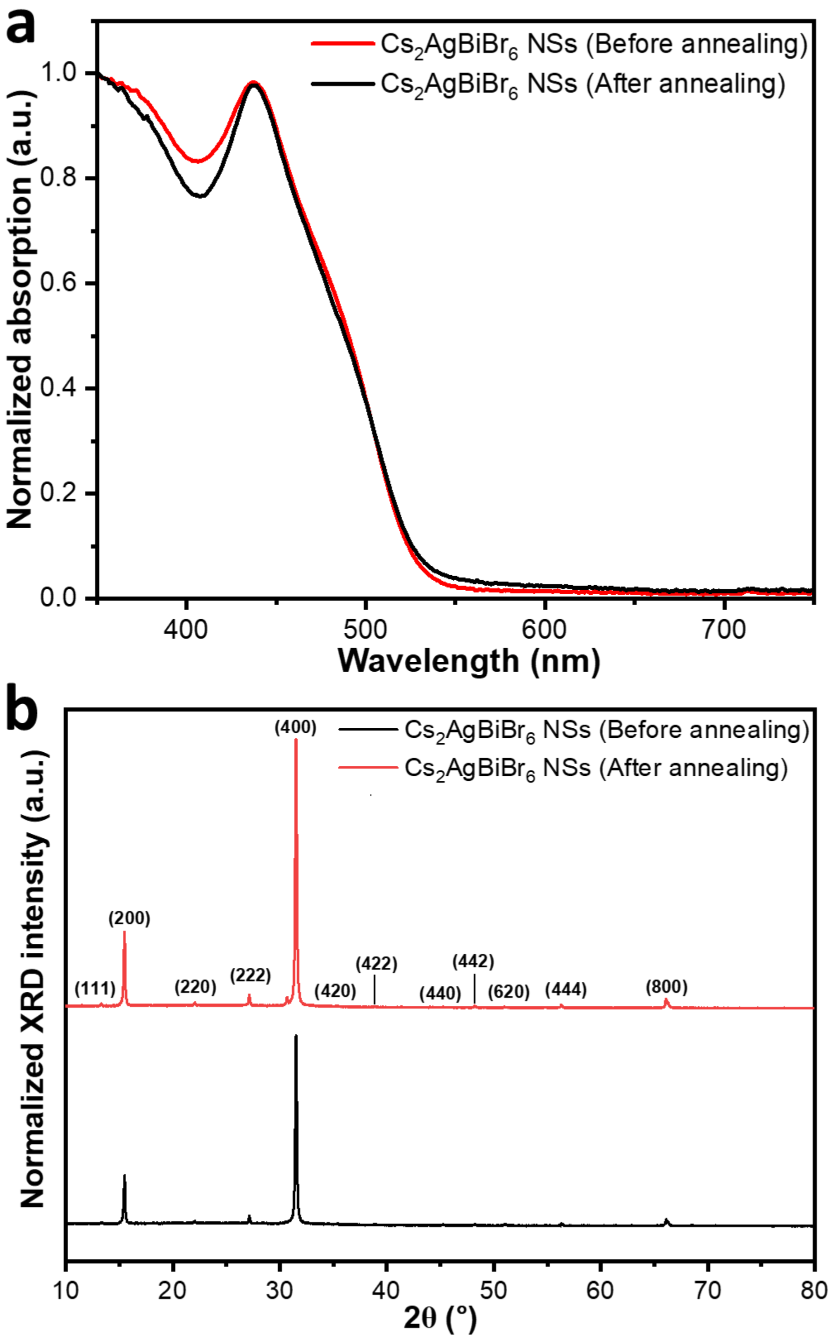

Figure S10. The colloidal $Cs_2AgBiBr_6$ NSs a) UV-vis absorption spectra of the calcined sample (200 °C) that was redispersed into toluene and that of the freshly prepared nanosheets, and b) XRD patterns of the annealed and the unannealed samples drop-casted on glass substrates.

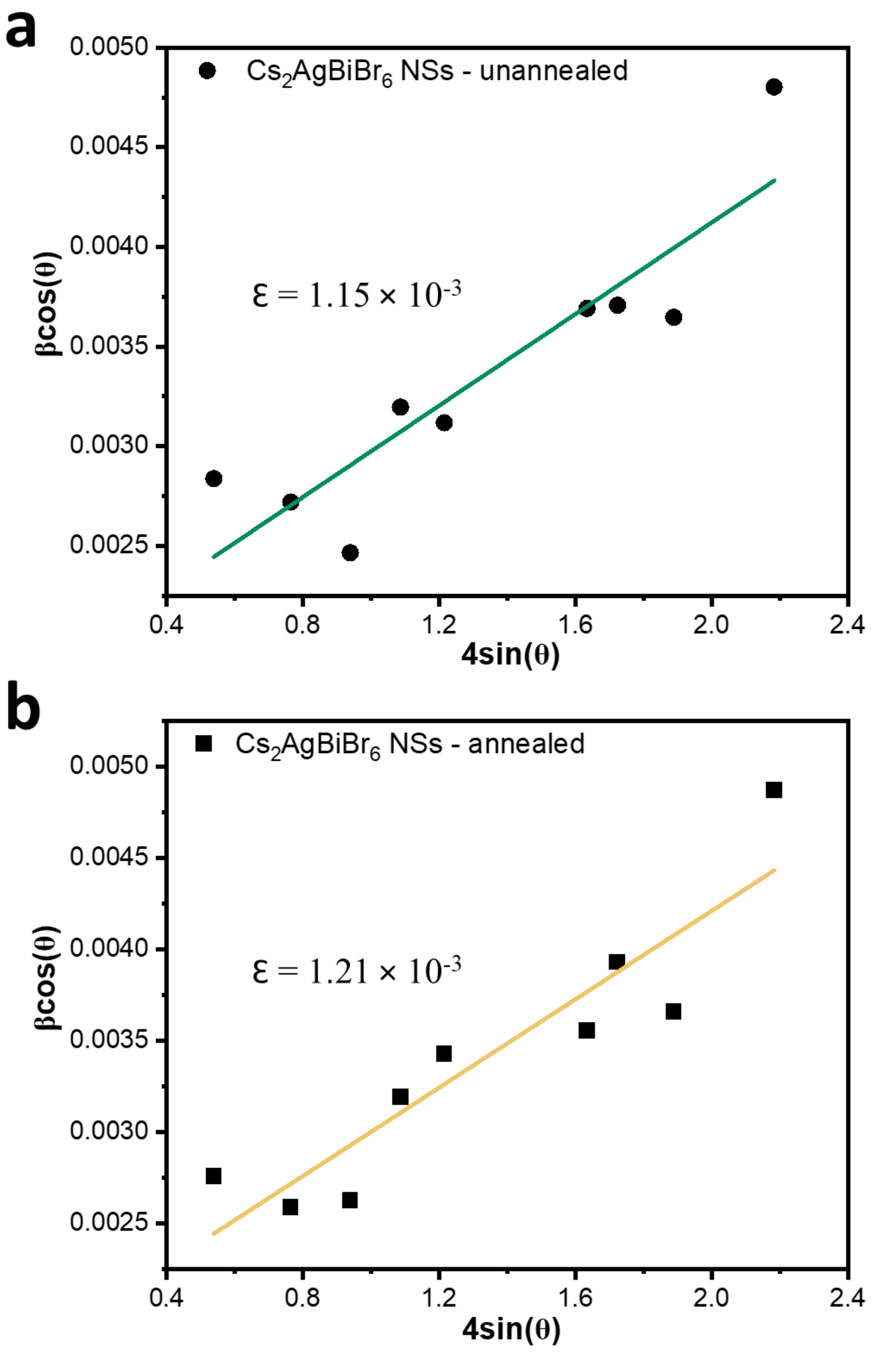

Figure S11. Williamson-Hall (W-H) plot of a) the annealed and b) the unannealed Cs2AgBiBr6 NSs samples evaluated from their XRD patterns. Following the Williamson-Hall approximation [2], the microstrain, Ɛ, was deduced for the annealed and unannealed samples from the linear fitting of the above W-H plots.

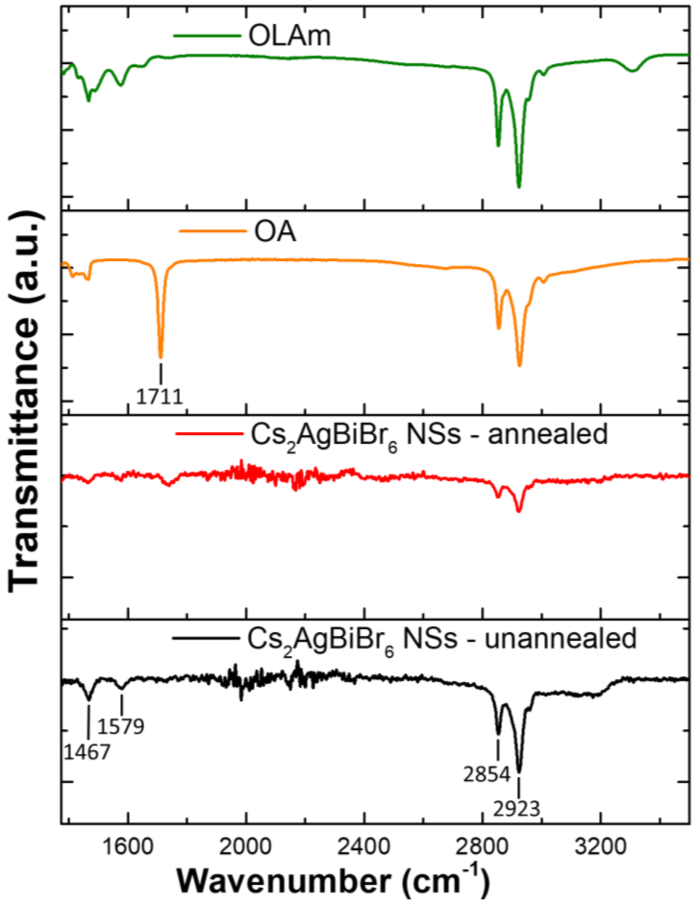

Figure S12. The FTIR spectra of unannealed and annealed $Cs_2AgBiBr_6$ NSs samples, including the spectra of pure OLAm and OA for reference purposes.

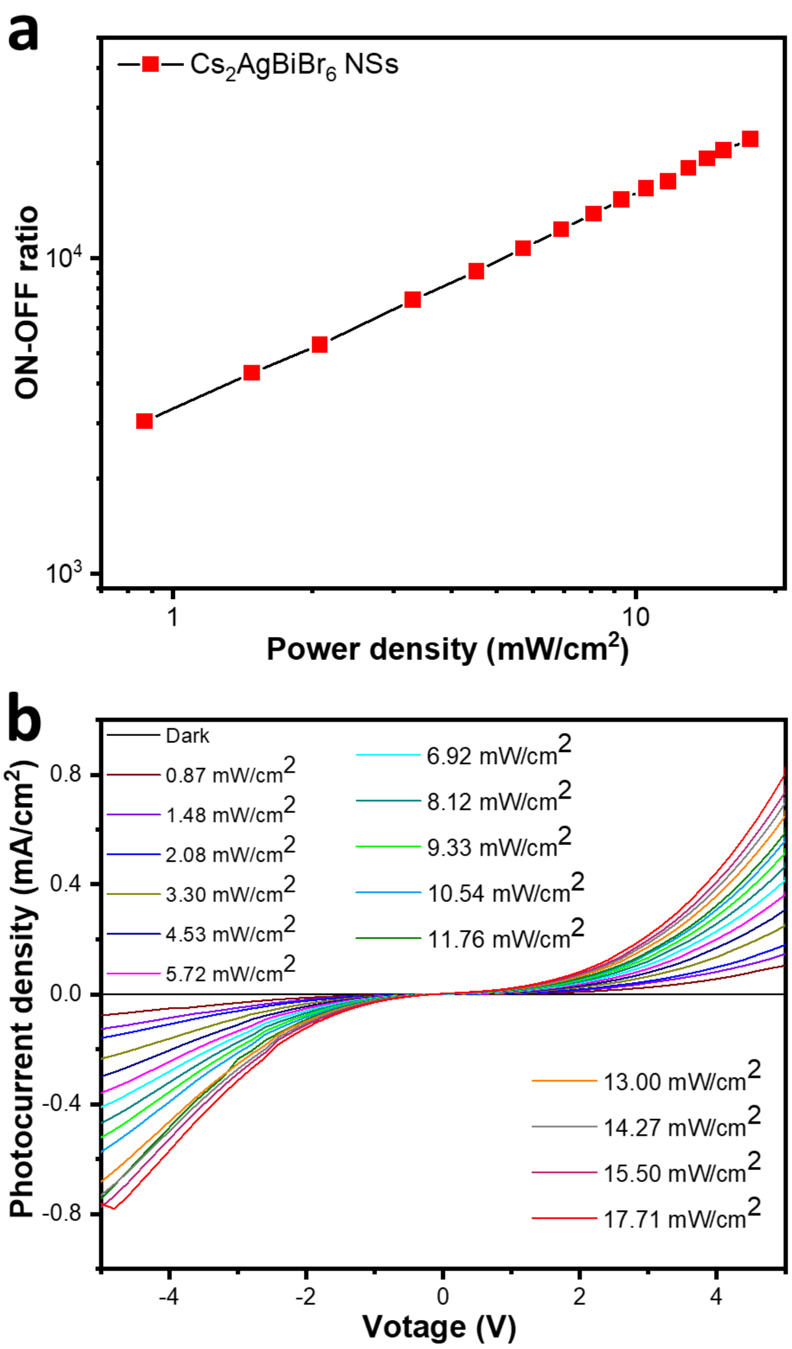

Figure S13. $Cs_2AgBiBr_6$ NSs photodetection device. a) on-off ratio at different illumination power densities and b) photocurrent density at various bias voltages (-5.0 to 5.0 V).

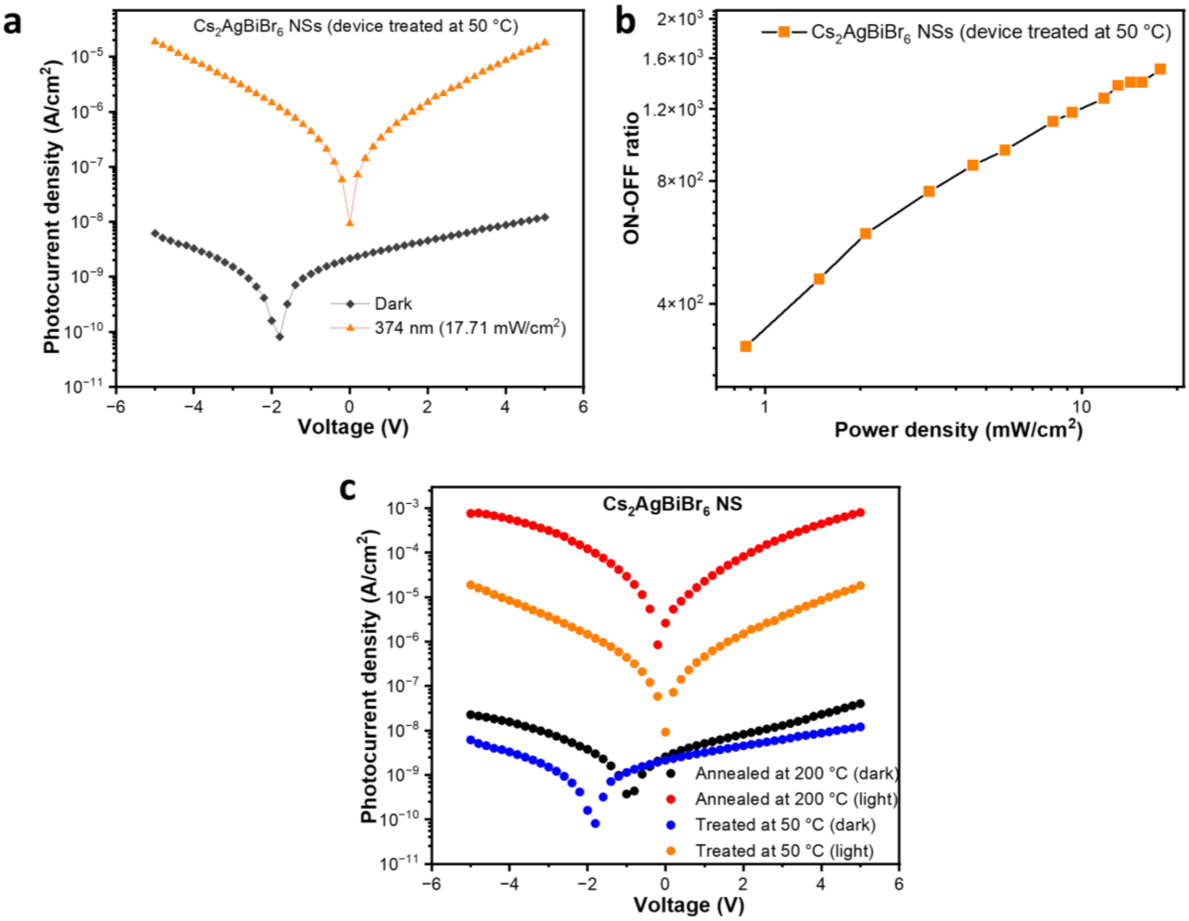

Figure S14. $Cs_2AgBiBr_6$ NSs photodetection device mildly treated at 50 ºC for 30 min. a) current under dark and illumination conditions within a voltage bias range of -5.0 to 5.0 V, and b) the on-off ratio of the device at various voltages: c) presents a comparison of current response for photodetectors treated at 50 ºC and annealed at 200 ºC under dark and illumination (374 nm laser, 17.1 mW/cm^2) conditions.

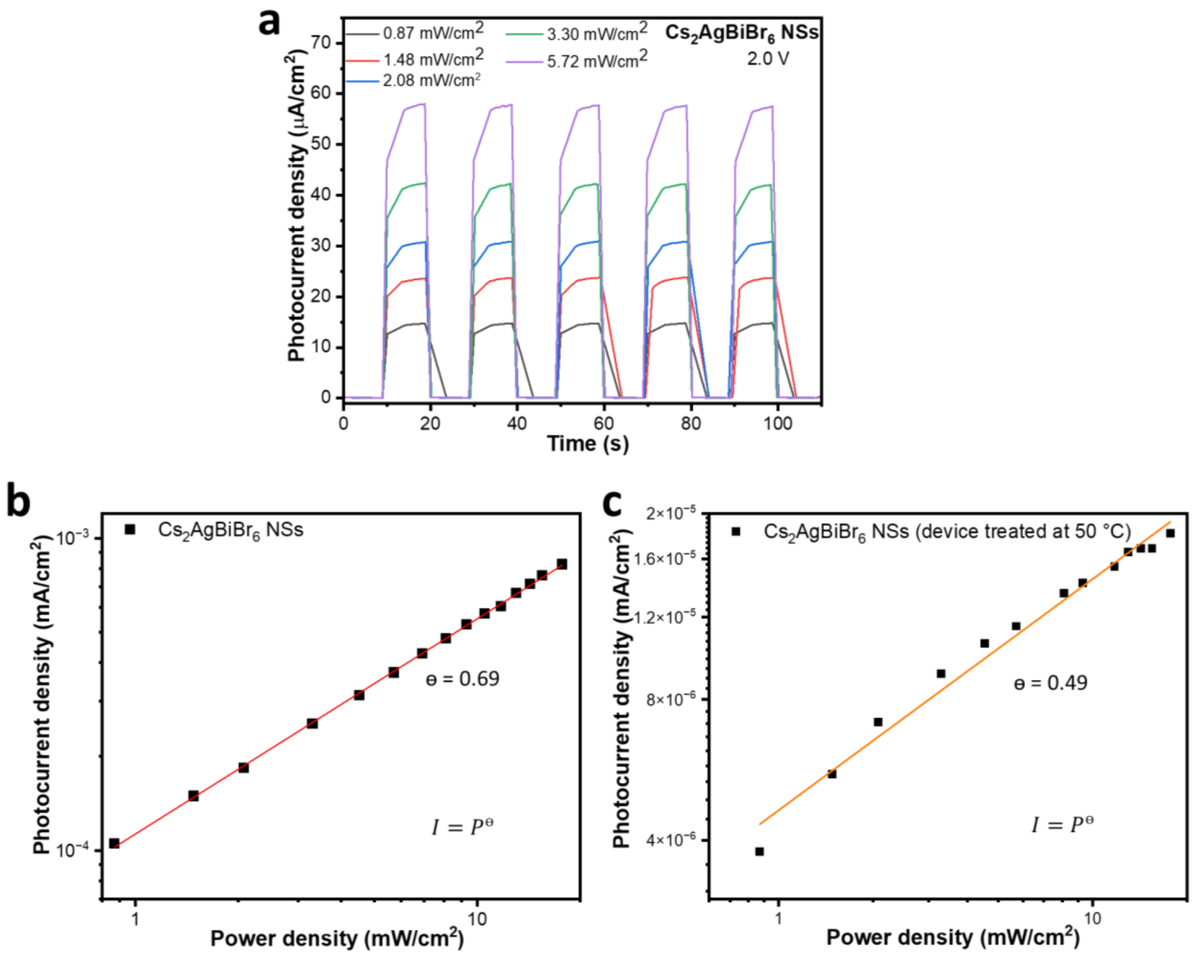

Figure S15. a) transient photocurrent density at different illumination power densities and constant voltage of 2.0 V, and b) and c) show the plot of photocurrent against incident power densities, fitted to the power law for the device annealed at 200 °C and mildly treated at 30 °C, respectively.

SI3

Considering short noise as the dominant source of noise in the photodetection device, the noise equivalent power (NEP) is calculated using the relations given in Equations S1 and S2:

$$\text{NEP} = \frac{\sqrt{(i_{noise})^2}}{R} = \frac{i_{noise}}{R} \quad \text{S1}$$

$$i_{noise} = i_{s,n} = \sqrt{2eI_{dark}B} \quad \text{S2}$$

where i_{noise} is the total noise current in the device, R represents the responsivity, I_{dark} is the dark current, e is the electronic charge, and B denotes the bandwidth of the equipment.

Generally, where the active area of the photodetector, A, is defined, the detectivity, D, is related to NEP by the formula given in Equation S3:

$$D = \frac{(AB)^{1/2}}{\text{NEP}} \quad \text{S3}$$

$$D = \frac{R}{\sqrt{2e(I_{dark}/A)}} \quad \text{S4}$$

Considering Equations S1, S2, and S3 above, D can be expressed as given in Equation S4 (the same as Equation 4 in the revised version of the manuscript), which was used in calculating the detectivity reported for the photodetection devices reported in this work [3, 4].

References

[1] S. Dror, S. Khalfin, N. Veber, A. Lang, Y. Kauffmann, M. Koifman Khristosov, R. Shechter, B. Pokroy, I.E. Castelli, and Y. Bekenstein, "Transformations of 2D to 3D Double-Perovskite Nanoplates of $Cs_2AgBiBr_6$ Composition," *Chemistry of Materials* 35 (2023): 1363-1372.

[2] G. Williamson and W. Hall, "X-ray line broadening from filed aluminium and wolfram," *Acta Metallurgica* 1 (1953): 22-31.

[3] F. Fang, H. Li, S. Fang, B. Zhou, F. Huang, C. Ma, Y. Wan, S. Jiang, Y. Wang, and B. Tian, "2D $Cs_2AgBiBr_6$ with boosted light–matter interaction for high-performance photodetectors," *Advanced Optical Materials* 9 (2021): 2001930.

[4] Y. Shan, W. Cui, Y. Zhou, N. Li, X. Wang, and B. Cao, "Lead-free perovskite $Cs_2AgBiBr_6$ epitaxial thin films for high-performance and air-stable photodetectors," *Journal of Materials Chemistry C* 13 (2025): 9072-9082.
